# CYTH4 Facilitates Renal Cell Carcinoma via Enhancing Proliferation and Likely Immune Evasion

**DOI:** 10.3390/biom16060923

**Published:** 2026-06-22

**Authors:** Ying Dong, Yingying Su, Damu Tang

**Affiliations:** 1 Department of Medicine, McMaster University, Hamilton, ON L8S 4K1, Canada; dongy87@mcmaster.ca (Y.D.); suy36@mcmaster.ca (Y.S.); 2 The Research Institute of St Joe’s Hamilton, St Joseph’s Hospital, Hamilton, ON L8N 4A6, Canada

**Keywords:** CYTH4, renal cell carcinoma, xenografts, immune evasion, overall survival

## Abstract

Cytohesin-4 (CYTH4), an ARF guanine nucleotide exchange factor, remains unknown in RCC pathogenesis. We report that CYTH4 was dramatically upregulated in clear cell renal cell carcinoma (ccRCC) and following ccRCC progression. CYTH4 was strongly associated with ccRCC’s immune-suppressive features and stratified ccRCC poor outcome. From CYTH4’s network/NW, a multigene panel, SigCYTH4NW, was derived. In retrospective studies, (1) SigCYTH4NW effectively predicted ccRCC’s inferior prognosis, was strongly associated with the well-validated poor risk ccB signature in four independent ccRCC cohorts (n = 1132), was significantly upregulated in ccB compared to ccA (favorable risk) tumors, was robustly correlated with an immune checkpoint signature (SigIC), and was predominantly expressed in tumor-associated macrophages, and (2) SigCYTH4NW effectively predicted poor prognosis and correlated with SigIC across 21 other cancer types. CYTH4 was expressed at low levels in 786-0 ccRCC cells; its stable expression promoted 786-0 cell proliferation in vitro and xenograft formation in vivo. CYTH4 bound PPP1R9B, which maintains pRb’s hypophosphorylation. 786-0 CYTH4 cells displayed intensive pRb hyperphosphorylation, suggesting that CYTH4 enhances cell proliferation partially by pRb inhibition. Gene expression profiling by RNA-seq revealed a 786-0 CYTH4 network that was relevant to primary ccRCC, particularly in the aspect of immune evasion. Collectively, this study supports CYTH4’s promoting ccRCC.

## 1. Introduction

Clear cell renal cell carcinoma (ccRCC) is the most common type of kidney cancer, accounting for 70–75% of cases, followed by papillary RCC (10–16%), chromophobe RCC (6%), and collecting duct carcinoma (0.5%) [1]. ccRCC is the top aggressive subtype and the predominant cause of kidney cancer-related mortality. The disease has a recurrence rate of approximately 20–40%, and 5–10% of patients progress to metastatic lethal ccRCC [2]. The mechanisms underlying ccRCC progression have been intensively investigated, leading to the discovery of the von Hippel–Lindau (VHL) hypoxia-inducible factor (HIF) axis as the primary driver of ccRCC oncogenesis and progression [3,4,5]. As a result of these molecular alterations, ccRCC features hypoxia [6], metabolic reprogramming towards aerobic glycolysis (the Warburg effect) [6,7], and an immune-infiltrated but immunosuppressive microenvironment [8]. These features facilitate the management of ccRCC, as evidenced by the utilization of immune checkpoint blockade and targeted therapies with tyrosine kinase inhibitors to vascular endothelial growth factor receptor (VEGFR) in treating metastatic ccRCC [9]. Nonetheless, these standard-of-care treatments are associated with limited effectiveness, which reflects our incomplete understanding of ccRCC’s etiology.

Cytohesin-4 (CYTH4) or PSCD4 is a member of the cytohesin family—CYTH1, CYTH2, CYTH3, and CYTH4—of ADP-ribosylation factor (ARF) guanine nucleotide exchange factors (GEFs) [10,11,12]. The family members contain typical structure domains: an N-terminal coil–coil (CC) domain mediating protein–protein interaction, a central Sec7 GEF catalytic motif, and a C-terminal pleckstrin homology (PH) plus a polybasic (PB) helix responsible for membrane recruitment [12,13,14,15]. These domains play integrated roles in cytohesins’ activities [12]. Cytohesins regulate actin cytoskeleton dynamics and membrane trafficking [16]. Apparently, these activities contribute to cell proliferation, motility, and invasion, implying the relevance of cytohesins to tumorigenesis. Notably, there is limited evidence of CYTH4 being relevant in ovarian cancer [17], breast cancer [18], acute myeloid leukemia [19], and melanoma [20]. However, its involvement in ccRCC remains to be investigated.

We report the first evidence of CYTH4-derived promotion of RCC tumorigenesis and progression. The CYTH4 protein is expressed in RCC tumor cells and is located in the cell membrane, consistent with its recruitment to plasma membranes to activate ARF6 [12,19,21]. CYTH4 is upregulated in ccRCC compared to normal tissues and advanced ccRCCs. CYTH4 promoted 786-0 cells to form colonies in vitro and xenografts in immunocompromised mice. It altered gene expression in 786-0 CYTH4 tumors characterized by proliferation and immune evasion. These features were likely presented in the multigene panel SigCYTH4NW, which was derived from CYTH4’s gene expression network. Both CYTH4 and SigCYTH4NW are robustly associated with an established immune checkpoint signature (SigIC) [22]. In retrospective investigations, SigCYTH4NW effectively stratified poor prognosis in ccRCC and 21 other cancer types with more than 6000 patients. Of importance, SigCYTH4NW risk scores display comparable hazard ratios (HRs) in assessing the risk of poor prognosis across these cancer types, supporting the important influence of the biological processes presented in SigCYTH4NW on a cancer’s aggressiveness. Notably, SigCYTH4NW displays robust correlation with an immune checkpoint-focused gene panel, SigIC [22], in multiple ccRCC cohorts and across 21 cancer types in retrospective analyses, suggesting that SigCYTH4NW predicts poor prognosis in ccRCC and across 21 cancer types, likely via its correlation with a cancer’s immune evasion features.

## 2. Materials and Methods

### 2.1. Cell Lines, Plasmids, and Retrovirus Infection

The 786-O ccRCC cell line was purchased from ATCC (Manassas, VA, USA) and cultured in RPMI1640 (Gibco, Carlsbad, CA, USA) under conditions with 1% penicillin–streptomycin (Gibco, Carlsbad, CA, USA) and 10% fetal bovine serum (Life Technologies, Burlington, ON, USA). Cell lines were routinely checked for mycoplasma contamination using a PCR kit (Applied Biological Materials, Richmond, BC, Canada, cat no.: G238). The coding sequence of CYTH4 was amplified by polymerase chain reaction (PCR) using complementary DNA (cDNA) as a template, which was reverse transcribed from total RNA isolated from THP1 cells. PCR was performed using the forward primer 5′-TAGCGCTACCGGACTCAGATCTATGGCCCAGAAGGAGAAGAG-3′ and the reverse primer 5′-CCTTAATGGCCTAACGAATTCTCACTGCTTGVTGGCAATCTTCT-3′. The amplified fragment was then digested with BglII (Invitrogen, Waltham, MA, USA) and EcoRI (Invitrogen, Waltham, MA, USA) and ligated into the MSCV IRES Luciferase (Addgene, Watertown, NY, USA, #18760) vector digested with the same enzymes. The IRES luciferase and CYTH4-IRES-Luciferase were further digested with BglIIand ClaI (Invitrogen, Waltham, MA, USA) and ligated into pLPCX vector. The resulting construct was verified by Sanger sequencing. The pLPCX-IRES-Luciferase or pLPCX-CYTH4-IRES-Luciferase was co-transfected with packing plasmids pGP and pVSV-G through the calcium phosphate transfection method. Viral supernatants were collected at 48 and 72 h post-transfection, filtered through a 0.45 μm membrane and used to infect 786-O cells. Stably transduced cells were selected with puromycin (2 μg/mL, Sigma Aldrich, Oakville, ON, Canada) for 7–14 days. CYTH4 overexpression was confirmed by Western blotting.

### 2.2. Colony Formation Assay

A colony formation assay was conducted by seeding cells in six-well plates with 100, 500, and 1000 786-0 cells. Colonies were fixed with fixation buffer (2% formaldehyde, Sigma Aldrich, Oakville, ON, Canada) and stained with crystal violet (0.5%, Sigma Aldrich, Oakville, ON, Canada) after being cultured for 2 weeks. Colony numbers were counted and analyzed.

### 2.3. Co-Immunoprecipitation (Co-IP) and Western Blotting

Cells were harvested and lysed in ice-cold lysis buffer (20 mM Tris (pH 7.4), 150 mM NaCl, 1 mM EDTA, 1 mM EGTA, 1% Triton X-100, 25 mM sodium pyrophosphate, 1 mM NaF, 1 mM β-glycerophosphate, 0.1 mM sodium orthovanadate, 1 mM PMSF, 2 μg/mL leupeptin and 10 μg/mL aprotinin) for 30 min on ice. Lysates were cleared by centrifugation at 12,000× *g* for 15 min at 4 °C, and protein concentration was determined by BCA assay (Biorad, Hercules, CA, USA).

For immunoprecipitation, 1 mg of total protein was incubated with 1 µg specific antibody (anti-CYTH4 FabGennix, Frisco, TX, USA; anti-PPP1R9B (Proteintech, Rosemont, IL, USA) or IgG (Cell Signalling, Danvers, MA, USA) plus Protein G agarose beads (Invitrogen, Waltham, MA, USA) overnight with rotation at 4 °C. Beads were washed three times with buffer containing 0 mM Tris (pH 7.5), 100 mM NaCl, 1.5 mM EGTA and 0.1% Triton X-100, and bound proteins were eluted by boiling in 2× SDS loading buffer for 10 min.

Eluted proteins were separated by SDS-PAGE and transferred to a PVDF membrane (Millipore, Oakville, ON, Canada). The membrane was blocked with 5% non-fat milk in TBST for 1 h at room temperature, followed by incubation with primary antibody against CYTH4 (Origene, Rockville, MD, USA, 1:1000) or PPP1R9B (Novus, Oakville, ON, Canada, 1:200) overnight at 4 °C. After washing, the membrane was incubated with HRP-conjugated secondary antibody (Biorad, Hercules, FL, USA, 1:3000) for 1 h at room temperature, and signals were detected using an enhanced chemiluminescence (ECL) substrate (Millipore, Oakville, ON, Canada). Input samples (5–10% of total lysate) were included as loading controls.

### 2.4. Immunofluorescence Staining (IF)

Cells cultured on 8-well chamber slides (Corning Life Sciences, New York, NY, USA) were fixed in 4% formaldehyde (Sigma Aldrich, Oakville, ON, Canada) for 15 min, and non-specific binding sites were blocked with PBS containing 1% BSA and 10% normal donkey serum (Vector Laboratories, Burlington, ON, Canada) for 1 h, followed by the addition of a 1:1 mixture of two primary antibodies, CYTH4 (Proteintech, Rosemont, IL, USA, 1:200) and PPP1R9B (Novus, Toronto, ON, Canada, 1:200), for 45 min at room temperature. Following 3  ×  5 min washes with PBS, slides were incubated with a mixture of two secondary antibodies, FITC Donkey anti-rabbit IgG (Cell Signaling, Danvers, MA, USA, 1:200) and Sheep IgG NorthernLights™ NL557 Donkey anti-Sheep igG (Novus, Toronto, ON, Canada, 1:200), for 30 min at room temperature. Slides were mounted with VECTASHIELD anti-fade mounting medium with DAPI (Vector Laboratories, Burlington, ON, Canada). Images were captured in 24 h with a fluorescence microscope (Axiovert 200; Carl Zeiss, Oberkochen, Germany). Quantification of staining was performed using Image J2 (v.2.16.0).

### 2.5. Xenograft Tumor Formation and Luminescent Imaging

786-0 EV and 786-0 CYTH4 cells were suspended in 0.1 mL RPMI/Matrigel (Corning Life Sciences, New York, NY, USA) mixture with a 1:1 volume and implanted subcutaneously into the left flank of 8-week-old non-obese diabetic/severe combined immunodeficiency (NOD/SCID) male mice (The Jackson Labtory, Bar Harbor, ME, USA). The mice were monitored post-injection of cancer cells through observation and palpation. The size of the tumors was measured every week by luminescent imaging. Briefly, the mice were injected with D-Luciferin (150 mg Luciferin/kg, GoldBio, St. Louis, MO, USA) in PBS solution intra-peritoneally. After 15 min the mice were imaged with a Bruker Imaging (Billerica, MA, USA) machine under anesthesia. Bioluminescent imaging of the tumor as well as white light imaging of each mouse was obtained. Tumor volumes were quantified by luminescence imaging. The mice were euthanized when the tumor volume reached 1500 mm^3^ or reached humane endpoints, such as loss of 20% body weight or ulceration. The xenograft tumor, together with all the major organs, was photographed and collected. All tumors were cut in half, with one half fixed with 10% formalin (VWR, Mississauga, ON, Canada) and the other half stored in −80 °C. The formalin-fixed tissue was processed by the Department of Histology (St. Joseph’s Healthcare, Hamilton, ON, Canada) and embedded in paraffin (Thermo Fisher Scientific, Burlington, ON, Canada). All the animal experiments were performed according to the protocols approved by the McMaster University Animal Research Ethics Board (24-04).

### 2.6. Immunohistochemistry (IHC)

Xenograft tumors were paraffin-embedded and cut serially with a microtome (Leica, Concord, ON, Canada). Then they were de-paraffinized in 100% xylene, followed by 100% and 70% EtOH series. Antigen retrieval buffer was prepared with sodium citrate buffer (PH = 6) in a steamer for 20 min. CYTH4 (1:200, Proteintech, Rosemont, IL, USA) and KI67 (1:1000 Abcam, Walthan, MA, USA). Antibodies were incubated at 4 °C overnight. Secondary anti-rabbit antibodies (Vector Laboratories, Burlington, ON, Canada, 1:200), VECTASTAIN ABC and DAB solution (Vector Laboratories, Burlington, ON, Canada) were subsequently added to the slides and incubated following the manufacturer’s protocol. Washes were performed with 1× PBS and distilled water. Slides were counterstained with hematoxylin (Sigma Aldrich, Oakville, ON, Canada); image analysis was conducted with ImageScope software (v12.1Leica Microsystems Inc., Deerfield, IL, USA). Staining intensity scores were calculated as HScores using the formula [HScore = (%Positive) × (Intensity) + 1]. Statistical analysis was performed by Student’s *t*-test, and *p* < 0.05 was considered statistically significant.

### 2.7. RNA Sequencing Analysis

RNA extraction was performed with an miRNeasy Mini Kit (Qiagen, Toronto, ON, Canada, no. 217004) and enriched for poly(A) mRNA using NEBNext^®^ Poly(A) mRNA Magnetic Isolation Modules (New England Biolabs, Whitby, ON, Canada). Unique dual indexes were used for library preparation, followed by sequencing at the McMaster Genomics Facility using a paired-end 2 × 50 bp configuration on the Illumina NextSeq 2000 P2 flow cell, with 30 M clusters aimed per sample. Galaxy (https://usegalaxy.org/) (accessed on 11 February 2026) was used to analyze RNA-seq reads, with low-quality reads and adaptor sequences removed. Alignment with a human genomic sequence (hg38) and a mouse genomic sequence (mm10) was achieved with HISAT2; read counts were executed using the “Featurecounts” function. Differentially expressed genes (DEGs) were produced with DESeq2. KEGG analysis and GSEA (Gene Set Enrichment Analysis) were performed using Galaxy; the FGSEA (fast preranked GSEA) was used for GSEA analysis.

### 2.8. Semi-Quantitative Real-Time PCR

Total RNA was isolated from 786-0 EV and 786-0 CYTH4 tumorsusing Iso-RNA Lysis Reagent (5 PRIME, Austin, TX, USA). Reverse transcription was performed using Superscript IV (Thermo Fisher Scientific, Burlington, ON, Canada). Semi-quantitative real-time PCR was conducted with the ABI 7500 Fast Real-Time PCR System (Applied Biosystems, Foster, CA, USA) using SYBR-green (Thermo Fisher Scientific, Burlington, ON, Canada). All RT-qPCR primers were designed using NCBI Primer-BLAST (https://www.ncbi.nlm.nih.gov/tools/primer-blast, accessed on 10 January 2026) against human RefSeq mRNA sequences, specifically selecting primers that do not cross-react with murine transcripts. This ensured that gene expression measurements, including immune-related genes such as NOD2 and PRR7, reflected human tumor cell-intrinsic transcription rather than murine stromal contamination. Primer sequences are provided in Supplementary Table S1. Gene expression was normalized to ACTB, which demonstrated stable expression across all xenograft tumor samples. Relative expression was calculated using the ΔΔCt method. Statistical comparisons were performed using ΔCt values, which follow a normal distribution suitable for parametric testing, using an unpaired two-tailed Student’s *t*-test. Results are presented as fold changes (2^−ΔΔCt^) relative to EV controls. RT-qPCR was performed on five independent biological replicates per group (n = 5 xenograft tumors). Each measurement was performed in technical triplicate.

### 2.9. Programs and Websites

This study used the following programs: R2: Genomics Analysis and Visualization Platform (http://r2.amc.nl, http://r2platform.com, accessed on 12 January 2026), cBioPortal [23], UALCAN [24], Metascape [25], TIMER [26], and TISIDB [27]. The R glmnet (version 4.1-10), survival (version 3.8-3), Maxstat (version 0.7-26), and other packages were also utilized.

### 2.10. Molecular Docking Analysis

Protein–protein docking of CYTH4 and PPP1R9B was performed using GRAMM-X (http://gramm.compbio.ku.edu/, accessed on 8 January 2026) to characterize their potential interaction interface. Three-dimensional protein structures were retrieved from the Protein Data Bank (PDB; http://www.rcsb.org/). Protein–protein interactions were analyzed and visualized using PyMOL (v3.1.6.1) and PDBePISA (https://www.ebi.ac.uk/pdbe/pisa/, accessed on 8 January 2026).

### 2.11. Assignment of SigCYTH4NW Risk Scores to Individual Tumors

Coefficients for the 13 component genes of SigCYTH4NW were derived from multivariate Cox proportional hazard (Cox PH) regression analysis using the R survival package, with overall survival (OS) as the outcome. Individual tumor risk scores were then calculated as a linear combination of each gene’s expression weighted by its corresponding coefficient: Risk Score = Σ(coefi × Geneiexp), where coefi denotes the regression coefficient and Geneiexp denotes the normalized expression level of the gene.

### 2.12. Statistical Analysis

Kaplan–Meier survival analyses and logrank tests were conducted using the R Survival package and tools provided by cBioPortal. Cox regression analyses were performed using the R 4.4.1 survival package. ROC and precision–recall (PR) profiles were constructed using the PRROC package in R. Two-tailed Student’s *t*-test, one-way ANOVA, and two-way ANOVA were performed for statistical analysis of two and more than two groups respectively, with *p* < 0.05 considered statistically significant. Tukey’s test was performed for post hoc analysis. Statistical analysis was conducted using GraphPad Prism10 (v.10.5.0), and data were presented as the mean ± SEM/SD. A value of *p* < 0.05 was considered statistically significant.

## 3. Results

### 3.1. CYTH4 Promotes ccRCC

**Figure 1:**
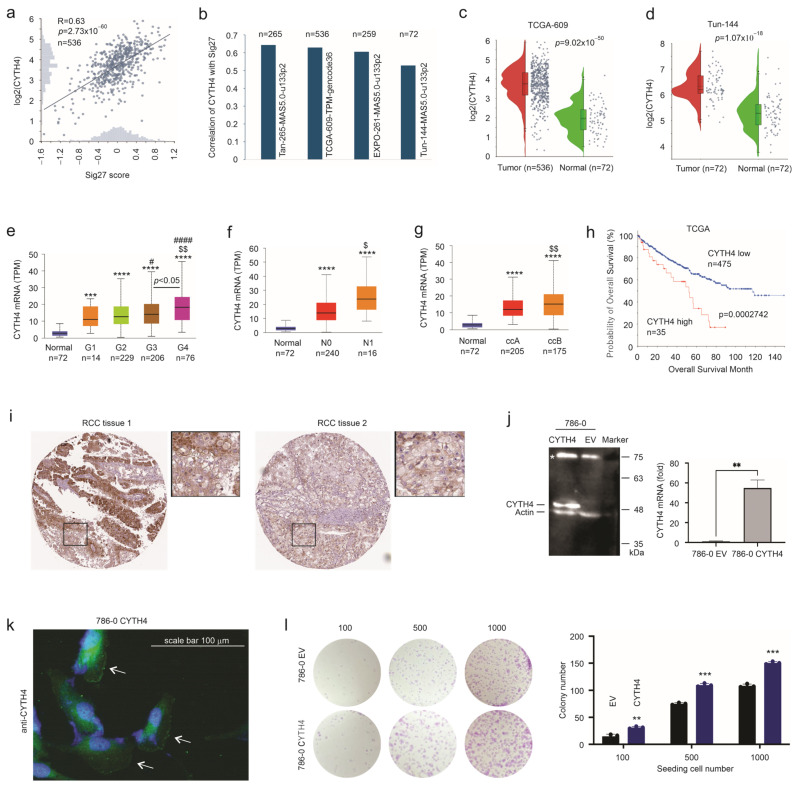
CYTH4 is relevant in RCC progression. (**a**,**b**) Correlations of CYTH4 expression with Sig27 metagene expression (score) in TCGA and the other three independent ccRCC cohorts. Analyses were performed using the R2: Genomics Analysis and Visualization Platform (http://r2.amc.nl, http://r2platform.com, accessed on 16 January 2026). (**c**,**d**) Upregulation of CYTH4 in ccRCC in the TCGA and Tun-144 datasets. Analysis was conducted using the R2 platform. (**e**–**g**) CYTH4 expression in the indicated clinical groups of ccRCC. *** *p* < 0.001 and **** *p* < 0.0001 compared to normal kidney tissues; # *p* < 0.05 and #### *p* < 0.0001 compared to G1 tumors (**e**); $ *p* < 0.05 and $$ *p* < 0.01 compared to G2 (**e**), N0 (**f**), and ccA (**g**). Analyses were performed using UALCAN (https://ualcan.path.uab.edu/index.html, accessed on 16 January 2026). (**h**) Kaplan–Meier overall survival analysis of ccRCC patients stratified by CYTH4 expression level. The cutoff point was estimated at 1.5 SD. Statistical analysis was performed using the log-rank test. (**i**) CYTH4 protein expression in RCC tissues. Images were downloaded from The Human Protein Atlas (The Human Protein Atlas, accessed on 8 November 2025). The marked region was 2-fold enlarged. (**j**) Western blot (left panel) and real-time PCR analysis (right panel) of CYTH4 expression in the indicated stable lines. Anti-CYTH4 antibody was used for Western blotting. Actin was used as the loading control for Western blotting and the housekeeping gene for real-time PCR. CYTH4 mRNA expression was normalized to actin and expressed in fold changes. Real-time PCR was performed in triplicate and repeated three times; mean ± SD was graphed. *: background band, ** *p* < 0.01 by 2-tailed Student’s *t*-test. (**k**) Immunofluorescence staining of CYTH4 in 786-0 CYTH4 cells using anti-CYTH4 antibody. Arrows indicate the potential leading edges which are CYTH4-positive. (**l**) 786-0 EV and 786-0 CYTH4 cells were seeded with the indicated density, cultured for 2 weeks, and stained with crystal violet. Experiments were repeated three times. Typical images from the same repeat and quantifications (mean ± SEM) are shown. ** *p* < 0.01 and *** *p* < 0.001 compared to the respective EV control analyzed using the 2-tailed Student’s *t*-test. The original WB images can be found in the supplementary materials.

CYTH4 was previously reported as an effective biomarker for assessing response to immune checkpoint therapy [20], consistent with its enrichment in immune tissues as well as its role in promoting cell migration and immunoregulation [16,28]. We recently reported a pan-RCC multigene panel Sig27 which predicts poor overall survival (OS) by capturing a tumor’s immunosuppressive properties [29]. Notably, CYTH4 correlates with Sig27 at high levels in four independent ccRCC datasets (Figure 1a,b). CYTH4 expression was significantly upregulated in ccRCC compared to normal kidney tissues in two independent cohorts (Figure 1c,d) and following ccRCC progression, as evidenced by increased CYTH4 expression in higher-grade ccRCC (Figure 1e) and lymph node metastasis (Figure 1f). Additionally, ccB tumors display a poor prognosis compared to ccA ccRCC [30]. CYTH4 is expressed at higher levels in ccB ccRCC (Figure 1g), and its expression stratifies ccRCC’s inferior OS (Figure 1h). The CYTH4 protein is known to be recruited to cell membranes to activate ARF6 and initiate downstream signaling [19,28]. CYTH4’s plasma membrane presence in RCC cells is highly suggested (Figure 1i), implying its functionality in enhancing ccRCC. Additionally, among the four cytohesin family members CYTH1, CYTH2, CYTH3, and CYTH4 [10,11,12], CYTH4 exhibits prominent alterations in ccRCC compared to normal kidney tissues (Supplementary Figure S1a), justifying our focus on CYTH4.

**Figure 2:**
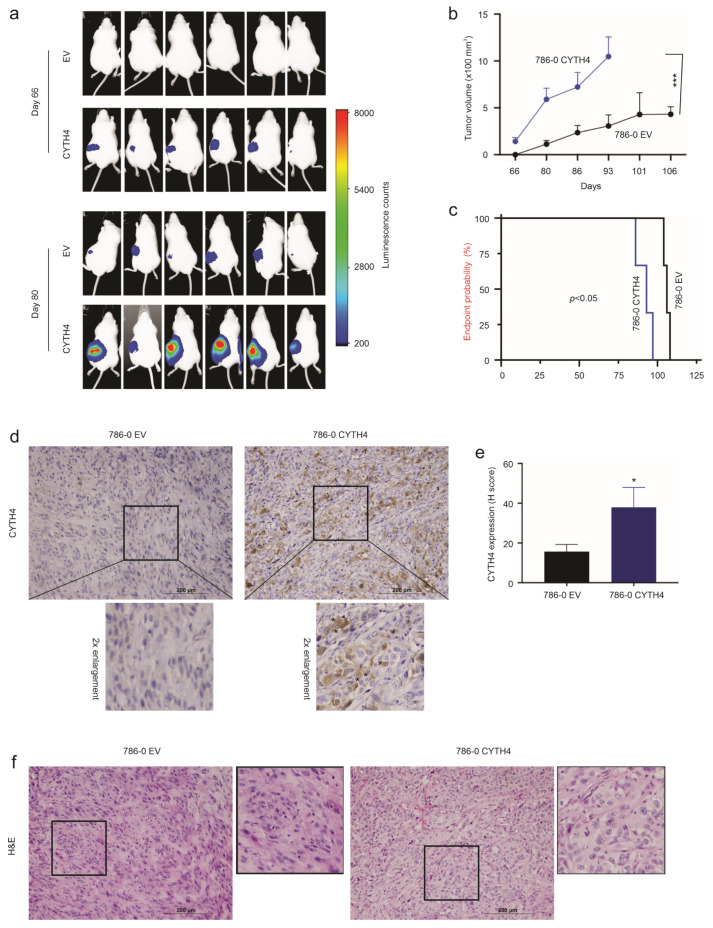
CYTH4 stimulates ccRCC tumor growth. (**a**) 786-0 EV-luciferase and 786-0 CYTH4-luciferase cells (5 × 10^6^) were subcutaneously implanted into NOD/SCID mice. Tumor growth was monitored by luminescence imaging. (**b**) Tumor volumes were quantified by luminescence imaging. *** *p* < 0.001 by two-way ANOVA. (**c**) Kaplan–Meier time-to-endpoint (reaching volume endpoint) curves of xenograft-bearing mice in the EV and CYTH4 groups. Statistical analysis was performed using the log-rank test. (**d**,**e**) Immunohistochemical staining for CYTH4 protein in the indicated xenografts (n = 5 per group). Typical images with 2× enlargement insets (**d**) and quantification (**e**) are presented. * *p* < 0.05 by 2-tailed Student’s *t*-test. Scale bars = 200 µm. (**f**) H&E staining of the indicated xenografts. The marked regions were enlarged in 2 folds.

To examine CYTH4’s functionality in ccRCC pathogenesis, we stably expressed an empty vector (EV) and CYTH4 in 786-0 ccRCC cells (Figure 1j), a VHL-null cell line [31]. Loss of VHL occurs in more than 90% of ccRCC cases [32,33]. 786-0 cells likely express low levels of CYTH4, given that its expression is largely restricted to hematopoietic cell types [28]. Indeed, endogenous CYTH4 was essentially undetectable under our Western blot conditions (Figure 1j), consistent with a recent report of a low level of CYTH4 in 786-0 cells [34]. This knowledge, together with the observed CYTH4 upregulation in primary ccRCC, justified its overexpression in 786-0 cells. Notably, CYTH4 was detected on cell membranes in the leading edges of 786-0 CYTH4 cells (Figure 1k), aligning well with CYTH4’s role in regulating actin cytoskeleton dynamics and consistent with its likely membrane presence observed in primary tumors (Figure 1i). However, in non-migrating 786-0 CYTH4 cells (based on their morphology), CYTH4 preferentially stayed in the cytosol (Supplementary Figure S1b), suggesting that its recruitment to the cell membrane is a dynamic process. 786-0 CYTH4 cells displayed increased colony formation ability in vitro (Figure 1l) and produced more aggressive tumors in NOD/SCID mice (Figure 2a–c). Elevations of CYTH4 expression in 786-0 CYTH4 tumors were confirmed (Figure 2d,e); its likely membrane presence was suggested (Figure 2d, see cells marked with an *). The 786-0 CYTH4 tumors display a more apparent stromal structure with more aggressive nuclear features compared to 786-0 EV xenografts (Figure 2f). Collectively, we provide comprehensive evidence demonstrating CYTH4 promoting ccRCC.

### 3.2. CYTH4 Alters ccRCC Gene Expression Affecting Tumor Immunity and Proliferation

**Figure 3:**
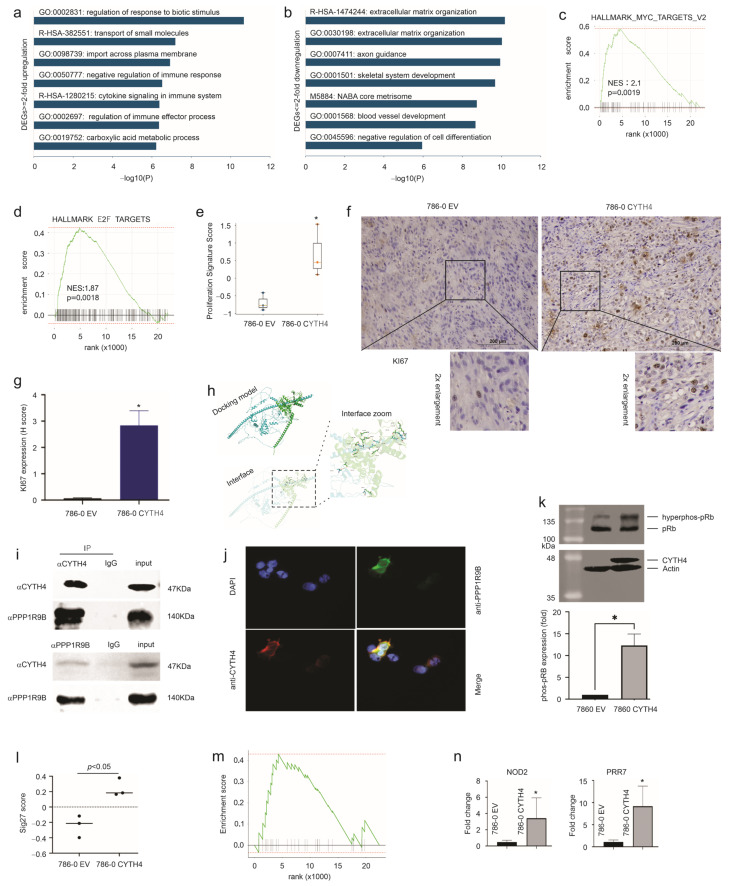
CYTH4 enhances ccRCC proliferation. (**a***,***b**) The top 7 enriched processes of DEGs with ≥2-fold upregulation (**a**) and ≤2-fold downregulation in 786-0 CYTH4 tumors. Analyses were performed using Metascape [25]. (**c**,**d**) Gene Set Enrichment Analysis (GSEA) of hallmark gene sets using DESeq2 Wald statistics derived from tumor-cell RNA-seq reads. (**e**) Sample-level MYC/E2F proliferation scores. Sample-level signature scores were calculated based on leading-edge genes identified from GSEA. Leading-edge genes (Supplementary Table S6) from the Hallmark MYC Target (V1/V2) and E2F Target gene sets were combined to form the proliferation signature. For the signature, a score was computed per sample as the mean z-scored expression of the constituent genes, using DESeq2-normalized counts as input. Group differences were assessed by unpaired 2-tailed Student’s *t*-test (**f**,**g**). IHC staining for Ki67 in the indicated xenografts (n = 5 per group). Typical images with 2× enlargement insets (**f**) and quantification (**g**) are presented. * *p* < 0.05 by 2-tailed Student’s *t*-test. Scale bars = 200 µm. (**h**) Docking modeling the interaction between CYTH4 and PPP1R9B using Pymol (V 3.1.6.1). The binding interphase is highlighted and enlarged. (**i**) Co-IP of endogenous CYTH4 and PPP1R9B from 786-0 CYTH4 cells. Experiments were repeated three times; typical results from a single repeat are shown. (**j**) Co-localization of CYTH4 and PPP1R9B. 293T cells were transiently co-transfected with CYTH4 and PPP1R9B, followed by immunofluorescence staining. DAPI, individual image channels, and merged images are presented. (**k**) Hypophosphorylated pRb (pRb) and hyperphosphorylated pRb (hyperphos-pRb) in the indicated cells were determined using phosphor-Rb D20B2 antibody (Cell Signaling, 1:1000). Experiments were repeated three times; typical images and quantification are shown. * *p* < 0.05 by 2-tailed Student’s *t*-test. (**l**) Sample-level Sig27 score calculated using RNA-seq data as the mean standardized expression of Sig27 genes. Statistical analysis was performed using Welch’s two-tailed *t*-test. (**m**) Pre-ranked GSEA of the Sig27 gene set using DESeq2 Wald statistics. The enrichment score for the Sig27 gene set was NES = 1.38, and *p* = 0.01143. (**n**) Real-time PCR amplification of NOD2 and PRR7 in the indicated tumors (n = 5 per group). * *p* < 0.05 by 2-tailed Student’s *t*-test. The original WB images can be found in the supplementary materials.

To systematically examine the mechanisms utilized by CYTH4 in promoting ccRCC, we profiled gene expression in 786-0 EV and 786-0 CYTH4 tumors (n = 3 per group) using RNA sequencing. A set of differentially expressed genes (DEGs) (*p* < 0.05, fold ≥ |1.5|) were derived (Supplementary Figure S2; Supplementary Table S2). As expected, CYTH4 is an upregulated DEG (Supplementary Figure S2). Upregulated DEGs with a fold change > 2 were enriched in regulation of response to biotic stimulus and pathways relevant to immune regulations (Figure 3a). Biotic stimulus encompasses tumor-associated antigens, damage-associated molecular patterns (DAMPs), and microbiome-derived ligands [35,36,37]. The top enrichment in GO:0002831: regulation of response to biotic stimulus is related to innate and adaptive immune signaling [36] and thus highly relevant to ccRCC. The enrichment of innate immunity-related signaling in 786-0 CYTH4 tumors is consistent with innate immune cells remaining functional in NOD/SCID mice [38] and the critical role of innate immunity in tumorigenesis [39,40,41]. The enrichment of R-HSA-382551: transport of small molecules and GO:0098739: import across plasma membrane may capture the biology of renal tubule epithelial cells, the origin of ccRCC [42]. Of relevance, alterations in VHL loss and HIF activation—the primary driver of ccRCC oncogenesis and progression—upregulate plasma membrane transporters under hypoxia to enhance tumor cell survival and metabolic reprogramming [43]. Regarding the downregulated DEGs (fold change ≤ −2), they were enriched in extracellular matrix organization, negative regulation of cell differentiation, and others (Figure 3b). Downregulation of these pathways may facilitate ccRCC progression. Collectively, the systematic alterations in gene expression observed in CYTH4-related DEGs support its actions in promoting ccRCC.

To further support the above findings, we analyzed the transcriptome of 786-0 CYTH4 tumors and that of 786-0 EV xenografts using Gene Set Enrichment Analysis (GSEA). The HALLMARK_MYC_TARGETS-V2 and HALLMARK_E2F_TARGETS gene sets were enriched in 786-0 CYTH4 xenografts (Figure 3c,d). Given that both gene sets are proliferation-focused, we observed higher MYC-E2F proliferation signature—MKI67, TOP2A, MCM2, CCNB1, CDK1, and PCNA [44,45]—scores in 786-0 CYTH4 xenografts (Figure 3e) and increased KI67 protein expression in 786-0 CYTH4 tumors (Figure 3f,g). Taken together, the above evidence suggests that CYTH4 facilitates 786-0 ccRCC tumor growth in part via enhancing cell proliferation.

The proliferation aspect was further supported by the primary association of CYTH4 interacting proteins (Supplementary Figure S3a)—derived using the BioPlex platform [46,47]—with cell proliferation. These proteins are predominantly enriched in the CORUM:7555:KEOPS complex (Supplementary Figure S3b), which promotes cell cycle progression [48]. MIS18A [49] and CCDC120 [50] are essential for mitosis. We further analyzed PPP1R9B (Spinophilin, SNP or Neurabin-2) (Supplementary Figure S3a) for binding to CYTH4. A molecular interaction analysis predicted a stable interacting phase formed by CYTH4 and PPP1R9B (Figure 3h). The endogenous CYTH4-PPP1R9B complex was co-immunoprecipitated (Co-IP) via either CYTH4 or PPP1R9B from 786-0 CYTH4 cell lysates (Figure 3i); their co-localization was demonstrated in 293T and 786-0 CYTH4 cells (Figure 3j; Supplementary Figure S4). PPP1R9B is a regulatory subunit of protein phosphatase 1 (PP1) and contributes to the dephosphorylation of pRb (retinoblastoma protein) and thus cell cycle arrest [51]. In this regard, 786-0 CYTH4 cells showed a significant elevation of hyperphosphorylated pRb (Figure 3k), implying a potential mechanism for 786-0 CYTH4 cells producing more colonies in vitro (Figure 1l) and aggressive xenografts (Figure 2) with increased KI67 expression (Figure 3f) in vivo.

### 3.3. CYTH4 Affects Gene Expression Relevant to Tumor Immune Escape

CYTH4 displays an effective biomarker potential in assessing melanoma response to immune checkpoint therapy [20] and a high-level correlation with Sig27 that predicts ccRCC poor prognosis by capturing ccRCC’s immunosuppressive features [29]. Notably, the Sig27 metagene is expressed at an increased level in 786-0 CYTH4 tumors (Figure 3l); the Sig27 gene set is enriched in the 786-0 CYTH4 transcriptome (Figure 3m); and three component genes (*NOD2*, *HDAC9*, and *PRR7*) of Sig27 are present in CYTH4 DEGs (Supplementary Figure S5a). We confirmed NOD2 and PRR7 upregulation in 786-0 CYTH4 xenografts (Figure 3n). NOD2 is strongly associated with ccRCC’s immunosuppressive features [20]. Together with the enrichment of immune signaling in CYTH4 DEGs (Figure 3a), these findings imply a contribution of CYTH4 to ccRCC’s immunosuppressive properties.

We also analyzed murine gene expression profiles associated with the 786-0 EV and 786-0 CYTH4 tumors and observed limited enrichment in the HALLMARK gene sets related to coagulation, angiogenesis, and hypoxia (Supplementary Figure S5b), implying that tumors with CYTH4 overexpression exhibit altered immune and proliferation features through both ccRCC and stromal cells (Supplementary Figure S5c).

### 3.4. CYTH4 Network Robustly Predicts ccRCC’s Poor OS in Retrospective Studies

**Figure 4:**
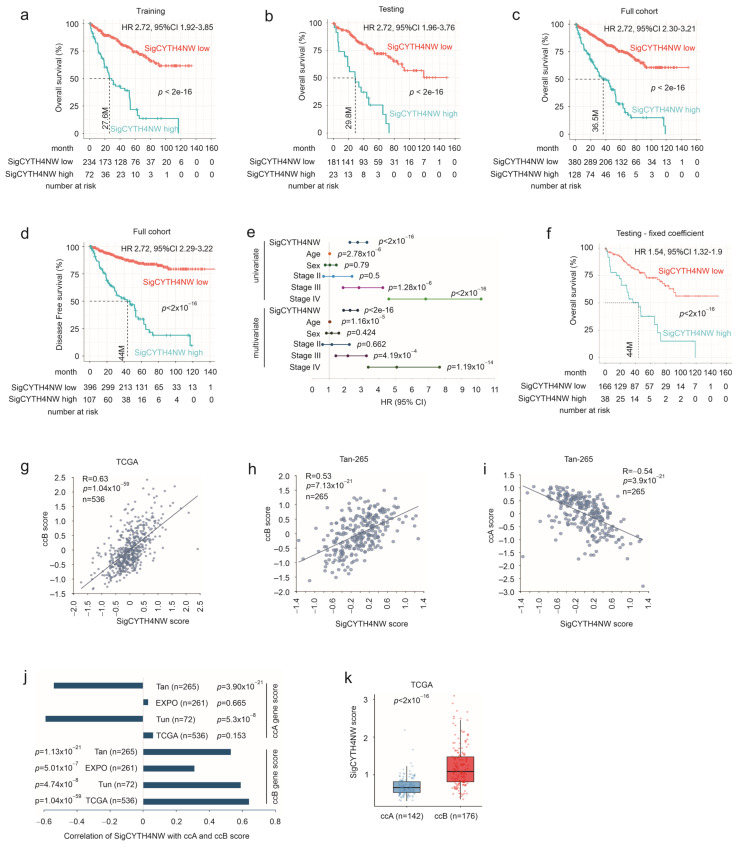
SigCYTH4NW stratifies ccRCC poor prognosis. (**a**–**c**) SigCYTH4NW risk scores were computed. Cutpoints were estimated using Maxstat. Kaplan–Meier survival curves and log-rank tests were performed using the R survival package. (**d**) SigCYTH4NW stratifies the recurrence risk of ccRCC. (**e**) Univariate and multivariate Cox analyses of SigCYTH4, age at diagnosis, sex, and stage in predicting OS probability were conducted using the TCGA Pancancer ccRCC cohort. (**f**) Stratification of poor OS in the testing sub-dataset using fixed coefficients obtained in the training sub-cohort. (**g**–**j**) Correlations of SigCYTH4NW with ccA and ccB in four independent ccRCC cohorts. Analyses were performed using the R2 platform. (**k**) Expression of SigCYTH4NW in ccA and ccB ccRCC subtypes.

Given the limitation of CYTH4 DEGs derived from a very small sample size of xenografts, we analyzed the CYTH4 network (NW) in the large TCGA ccRCC dataset. CYTH4 expression separates ccRCC within the TCGA PanCancer ccRCC dataset into high- and low-fatality risk groups (Figure 1h). We thus derived DEGs from both groups (*p* < 0.05 and fold change ≥ |2|), organized a dataset by retrieving their expression along with relevant clinical features, randomized the dataset into training (n = 304) and testing (n = 204) populations at a ratio of approximately 6:4, and performed a covariate selection to optimize a multigene panel for predicting OS probability using Elastic-net within the R glmnet package. A 13-gene panel (SigCYTH4NW) was obtained (Supplementary Table S3). We computed cohort-specific SigCYTH4NW risk scores by summing the weighted individual gene expressions using their Cox coefficients derived from a multivariate Cox model. SigCYTH4NW risk scores dramatically stratified poor OS in the training (Figure 4a), testing (Figure 4b), and full cohorts (Figure 4c), as well as disease-free survival (recurrence) (Figure 4d). SigCYTH4NW predicts poor OS independent of age, sex, and tumor stage (Figure 4e). We further assayed SigCYTH4NW biomarker potential in the testing sub-cohort using fixed coefficients obtained in the training sub-dataset; SigCYTH4 risk scores remain effective in the stratification of poor OS (Figure 4f), which argues against substantial overfitting.

To further address the overfitting potential, we determined SigCYTH4NW biomarker value by benchmarking against ClearCode34 signatures, a well-validated panel classifying ccRCC into ccA and ccB subtypes with favorable and poor prognoses, respectively [30,52]. The 34 genes of ClearCode34 consist of 24 ccA subtype genes and 10 ccB subtype genes [30,52]. Notably, SigCYTH4NW scores positively correlated with ccB scores at a high level in four independent cohorts: TCGA, Tan, Tun, and EXPO (Figure 4g–j). SigCYTH4NW displayed no or a negative correlation with the ccA panel in these datasets (Figure 4i,j). Furthermore, in the classified ccA/ccB subtypes [53], the SigCYTH4NW metagene was dramatically upregulated in ccB ccRCCs (Figure 4k). These observations collectively support that SigCYTH4NW is not associated with major overfitting.

**Figure 5:**
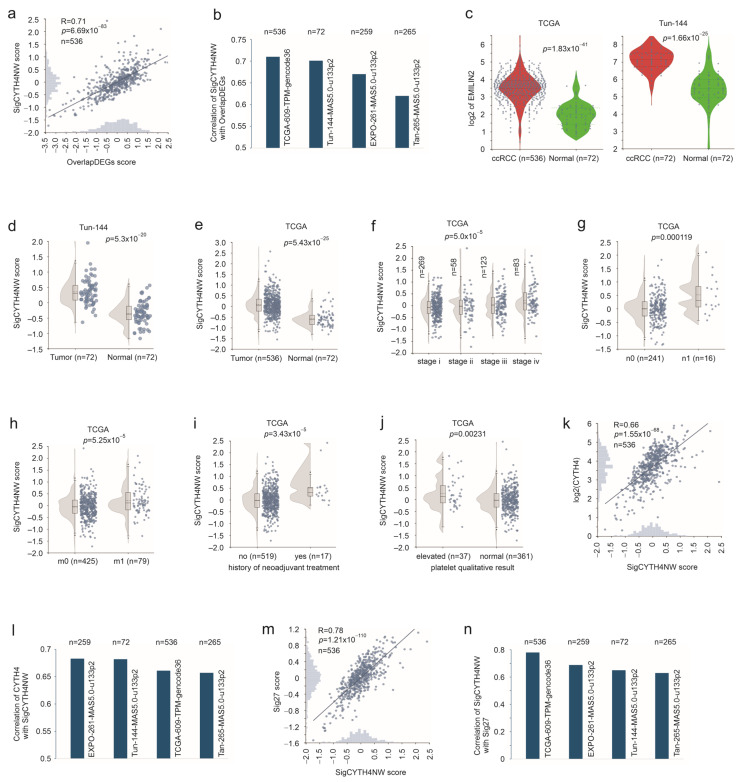
Characterization of SigCYTH4NW. (**a**,**b**) Correlations of SigCYTH4NW with OverlapDEGs in four independent ccRCC cohorts. Analyses were performed using the R2 platform. (**c**–**j**) Upregulation of EMILIN2 (**c**) as well as the SigCYTH4NW metagene (score) in tumor (**d**,**e**), stages (**f**), lymph node metastasis (**g**), distant metastasis (**h**), neoadjuvant treatment (**i**), and platelet count (**j**) in the indicated ccRCC datasets. Analyses were performed using the R2 platform. (**k**–**n**) Correlations of SigCYTH4NW with either CYTH4 (**k**,**l**) or Sig27 (**m**,**n**) in the indicated ccRCC cohorts. Analyses were performed using the R2 platform.

We used multiple approaches to validate SigCYTH4NW. Xenograft-derived DEGs contain 10 overlapping genes (Supplementary Figure S6a), with CYTH4 correlated genes obtained from TCGA ccRCC (Supplementary Table S4). Their upregulation in 786-0 CYTH4 tumors was confirmed (Supplementary Figure S6b). The overlapped 10-gene panel (OverlapDEGs) stratifies inferior OS of ccRCC (Supplementary Figure S6c). The unique feature of these 10 genes (except FMNL1) is their primary involvement in immune regulations (Supplementary Table S5). GBP5 (guanylate-binding protein 5) plays crucial roles in innate immunity [54,55]. LGALS9 is an established immune checkpoint in part owing to its expression in tumor cells and its promotion of cancer immune evasion [56]. These genes are primarily enriched in immune regulation, cell shape (relevant to cytoskeleton dynamics), and acute myeloid leukemia (Supplementary Figure S6d), aligning with the known activities of CYTH4 [19]. OverlapDEGs display high-level correlations with SigCYTH4NW in the TCGA and the other three independent ccRCC cohorts (Figure 5a,b). *EMILIN2* is a component gene of SigCYTH4NW (Supplementary Table S3), and, of importance, its increases occurred in ccRCC in two independent datasets (Figure 5c). Collectively, the above evidence supports a relationship between SigCYTH4NW and the gene expression of 786-0 CYTH4 tumors.

SigCYTH4NW scores or metagene expression were elevated in ccRCC compared to normal kidney tissues in two independent cohorts (Figure 5d,e), as well as in advanced-stage ccRCCs (Figure 5f) and lymph node and distant metastasis (Figure 5g,h). Previous neoadjuvant treatment and elevations in platelet counts were also associated with higher SigCYTH4NW scores (Figure 5i,j). The treatment pressure may drive ccRCC progression [57]; a high platelet count is an established risk marker of ccRCC [58,59]. SigCYTH4NW scores were highly correlated with CYTH4 expression in the TCGA dataset—the dataset from which SigCYTH4NW was derived (Figure 5k)—and in three additional independent cohorts (Figure 5l), with particularly strong correlations observed in both the EPOX-261 and Tun-144 cohorts (Figure 5l). Nonetheless, SigCYTH4NW exhibited superior prediction of poor OS compared to CYTH4 (Supplementary Figure S7). Additionally, SigCYTH4NW robustly correlated with Sig27—an established pan-RCC biomarker [29]—in four ccRCC cohorts (Figure 5m,n). Collectively, the above analyses provide solid validation of SigCYTH4NW as a novel biomarker of ccRCC.

### 3.5. SigCYTH4NW Predicts Poor OS in Papillary RCC (pRCC), Chromophobe RCC (chRCC), and Across a Panel of Cancer Types in Retrospective Datasets

**Figure 6:**
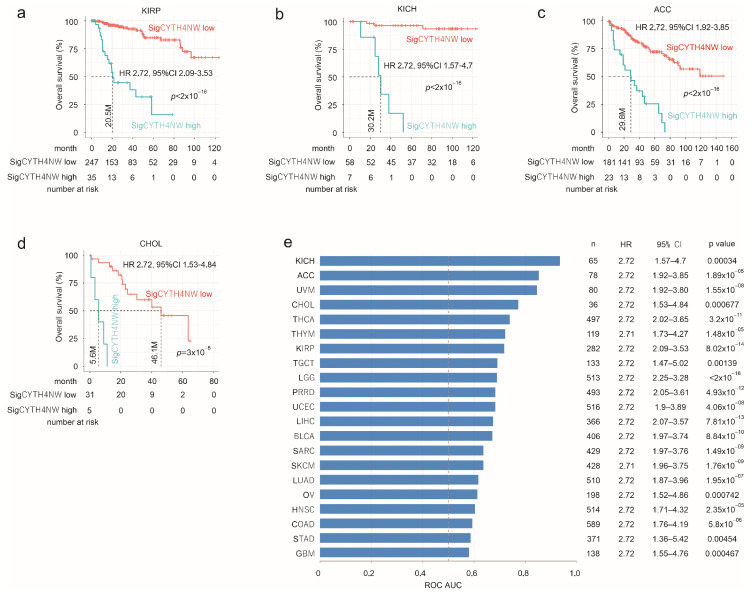
SigCYTH4NW predicts poor prognosis across 21 cancer types. (**a**–**d**) Cohort-specific SigCYTH4NW risk scores were calculated and used to construct Kaplan–Meier survival curves using the R survival package. A log-rank test was performed. (**e**) The AUC values of SigCYTH4NW in discriminating prognostic outcomes in the indicated cancer types. The sizes (n) of TCGA PanCancer datasets, HRs (hazard ratios), 95% CIs (confidence intervals), and *p* values of SigCYTH4NW risk scores in predicting poor prognosis are included. ACC: adrenocortical carcinoma; BLCA: bladder urothelial carcinoma; CHOL: cholandiocarcinoma; COAD: colon adenocarcinoma; GBM: glioblastoma multiforme; HNSC: head and neck squamous cell carcinoma; KICH: chromophobe renal cell carcinoma; KIRP: kidney renal papillary cell carcinoma; LGG: brain lower-grade glioma; LIHC: liver hepatocellular carcinoma; LUAD: lung adenocarcinoma; OV: ovarian serous cystadenocarcinoma; PRAD: prostate adenocarcinoma; SARC: sarcoma; SKCM: skin cutaneous melanoma; STAD: stomach adenocarcinoma; TGCT: testicular germ cell tumor; THCA: thyroid carcinoma; THYM: thymoma; and UVM: uveal melanoma.

We further validated SigCYTH4NW across other cancer types. The signature effectively stratifies poor OS in two other types of RCC—pRCC (Figure 6a) and chRCC (Figure 6b)—as well as across 19 other cancer types (Figure 6c–e). Notably, the risk stratification in ACC (Figure 6c), CHOL (Figure 6d), and other cancer types (Figure 6e) appears to be robust, providing additional and solid validation of SigCYTH4NW. Furthermore, SigCYTH4NW risk scores displayed comparable hazard ratios (HRs) in poor prognosis across 21 cancer types (Figure 6e), suggesting that the biological changes presented in SigCYTH4NW exert a comparable influence on tumor aggressiveness across other cancer types. In comparison, the variations in ROC-AUC across cancer types (Figure 6e) reflect cohort- and cancer-specific heterogenous factors which affect the prognostic event (cancer death), including cancer-specific survival heterogeneity, baseline hazard rates, event time and distribution, noise, treatment, and censoring pattern, as well as follow-up time.

### 3.6. SigCYTH4NW Associates with ccRCC’s Immunosuppressive Signaling in Retrospective Datasets

**Figure 7:**
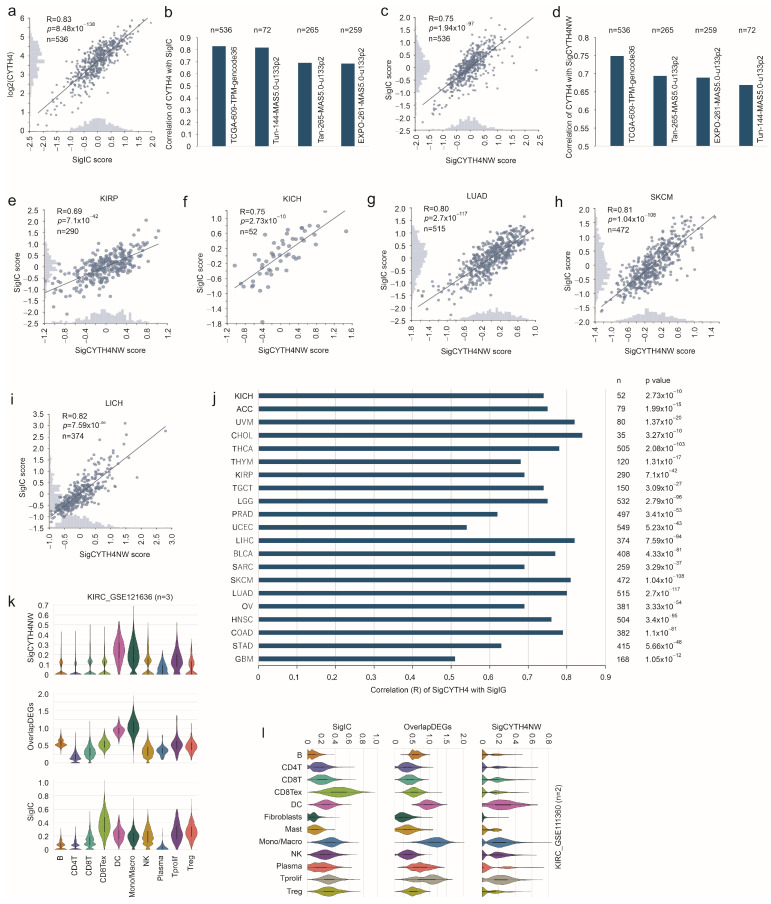
SigCYTH4NW correlates with SigIC across 21 cancer types. (**a**–**d**) Correlation of CYTH4 with SigIC and correlation of SigCYTH4NW with SigIC in the four independent ccRCC cohorts. (**e**–**j**) Correlation of SigCYTH4NW and SigIC in the indicated cancer types, including typical correlation graphs (**e**–**i**) and summary graph (**j**). All analyses were performed using the R2 platform. (**k**,**l**) Expression of SigCYTH4NW, OverlapDEGs, and SigIC in the indicated single-cell populations of ccRCC in two scRNA ccRCC datasets. Analyses were performed using the TISCH2 platform [64].

Given the role of CYTH4 in effectively predicting a cancer’s response to immune checkpoint therapy [20] and the high-level correlations between CYTH4 and SigCYTH4NW in four independent ccRCC datasets (Figure 5k,l), SigCYTH4NW may predict poor OS in RCC and multiple cancer types (Figure 6) via its association with the biological processes relevant to cancer immune evasion. Notably, both CYTH4 and SigCYTH4NW robustly correlate with SigIC—a 22-gene panel comprising established immune checkpoint genes that are strongly associated with cancer immune evasion [22]—in four independent ccRCC cohorts (Figure 7a–d). High-level correlations extend to two other types of RCC—pRCC (KIRP) (Figure 7e) and chRCC (KICH) (Figure 7f)—lung adenocarcinoma (LUAD) (Figure 7g), melanoma (SKCM) (Figure 7h), hepatocellular carcinoma (LIHC) (Figure 7i), and all the cancer types (Figure 7j) for which poor prognosis was significantly predicted by SigCYTH4NW (Figure 6e). Additionally, among single-cell (sc) populations of ccRCC, SigCYTH4NW was primarily expressed in tumor-associated macrophage (TAM) and dendritic cells (DCs) with clear presence in exhausted CD8+ T (CD8Tex) and Treg cells (Figure 7k,l). A similar pattern was also observed for overlap DEG expression (Figure 7k,l), providing additional support for the relevance of the CYTH4 network derived from primary ccRCCs to that originated from xenografts. As expected, SigIC is predominantly expressed in CD8Tex, Treg, and/or TAM cells (Figure 7k,l). CD8Tex, TAM, and Treg cells are well known for their major contributions to cancer immune evasion [60,61,62]. Tumor-derived signaling induces immune tolerance in DCs, thereby contributing to cancer immune escape [63]. Collectively, the above evidence aligns with the notion that SigCYTH4NW predicts poor OS across 22 cancer types, including ccRCC, possibly and in part via the immune evasion components present in SigCYTH4NW.

To further support the above concept, we obtained genes correlated with SigCYTH4NW metagene expression in four independent ccRCC datasets (TCGA, Tun-144, Tan-256, and EXPO-261). The positively correlated genes are commonly enriched in immune processes in these cohorts (Supplementary Figure S8), supporting SigCYTH4NW containing ccRCC’s immune-evasive components.

## 4. Discussion

Clear cell RCC remains one of the most lethal urological malignancies, which is in part attributed to its immune evasion properties. The tumor has one of the most abundant immune infiltrates among solid tumors [8], yet the level of infiltrated CD8+ T cells does not translate to a clinical response to PD1 inhibitor nivolumab therapy [65], the newly established standard of care in treating advanced ccRCC [66], highlighting the need to deepen our understanding of ccRCC etiology.

We report the first evidence of CYTH4 as a novel factor promoting ccRCC tumorigenesis and progression. CYTH4 expression shows a preference for hematopoietic and immune tissues [28], consistent with its recently detected oncogenic actions in acute myeloid leukemia [19] and as a biomarker predicting a solid cancer’s response to immune checkpoint therapy [20]. Notably, our research reveals clear CYTH4 expression in ccRCC tumor cells with a strong cell membrane presence (Figure 1i), an established functional site [19], suggesting acquisition of CYTH4 during ccRCC pathogenesis and progression. Of importance, acquisition of CYTH4 via its ectopic expression in 786-0 ccRCC cells promotes cells’ ability to produce tumors in NOD/SCID mice with potential membrane expression (Figure 2d), providing clinical and functional evidence of CYTH4 enhancing ccRCC. The membrane presence of CYTH4 appears to occur in migrating 786-0 cells in vitro, indicating that some unknown mechanisms regulate its membrane recruitment. Given that the C-terminal PH domain and PB helix mediate CYTH4 membrane recruitment [12,13,14,15], it is tempting to suggest that these mechanisms act on the C-terminal structures to induce CYTH4’s recruitment to the plasma membrane. Further investigations are needed to delineate these regulations. 786-0 CYTH4 tumors possess enriched stroma compared to 786-0 EV xenografts (Figure 2f), suggesting that CYTH4 may promote ccRCC in part via facilitating the communications between tumor cells and stromal cells. These communications may regulate CYTH4 expression in a feedback manner; for instance, CYTH4 was expressed at a higher level in 786-0 CYTH4 cells than in cell-produced tumors (comparing Figure 1j to Figure 2d,e). Given that tumor cells educate DCs to convert DCs from immunogenic to tolerogenic states [63], CYTH4 may facilitate this conversion. While the detailed mechanisms underlying CYTH4-promoted ccRCC progression remain unclear, evidence suggests the involvement of tumor immunity and proliferation.

The possibility that CYTH4 likely mediates promotion of ccRCC immune evasion is supported by (1) the upregulation of LGALS9—an established immune checkpoint facilitating immune suppression in part via its tumor cell expression [67], including ccRCC cells [68,69,70]—in 786-0 xenografts (Supplementary Figure S6b), (2) CYTH4’s high-level correlations with Sig27—a pan-RCC biomarker capturing RCC immune properties [29]—in multiple ccRCC cohorts (Figure 1a,b), (3) CYTH4’s strong correlations with SigIC—a multigene panel formed by 22 established immune checkpoints [22]—in four independent ccRCC cohorts (Figure 7a,b), (4) CYTH4’s prominent correlations with SigCYTH4NW (Figure 5k,l), and (5) the high-level correlation of SigCYTH4NW with SigIC not only in ccRCC but also across 21 other cancer types (Figure 7e–j). Additionally, the CYTH4 network derived from 786-0 xenografts is relevant to that obtained from primary ccRCCs, as evidenced by the presence of OverlapDEGs and the high-level correlations between SigCYTH4NW and OverlapDEGs (Figure 5a,b). Given the immune signaling focus of OverlapDEGs (Supplementary Table S5, Supplementary Figure S6), SigCYTH4NW’s correlation with OverlapDEGs provides additional support for SigCYTH4NW likely reflecting cancer’s immune evasion features. We are thus tempted to propose that the biomarker potential of SigCYTH4NW is partially attributable to its likely reflecting tumor immunity, implying SigCYTH4NW’s potential in assessing a cancer’s response to immune checkpoint therapy. Nonetheless, we would like to stress that the study solely utilized the retrospective ccRCC and pan-cancer datasets. In this regard, the observed associations of CYTH4 and SigCYTH4NW with the immunosuppressive features or immune evasion in ccRCC and pan-cancer remain preliminary, requiring validations in the future both functionally within experimental settings and clinically in prospective studies. Additionally, the prognostic values observed for SigCYTH4NW should be interpreted with caution. Its promising biomarker potential and the exploratory nature of the research thus call for prospective studies and clinical trials to validate SigCYTH4NW’s prognostic value in the future.

CYTH4 also facilitates ccRCC proliferation, as evidenced by the elevation of KI67 signaling in 786-0 CYTH4 tumors compared to 786-0 EV xenografts (Figure 3f). This activity might be partially attributed to CYTH4 binding to PPP1R9B, which inhibits cell cycle progression by maintaining pRb’s active status [51]. pRb phosphorylation is regulated by dynamic mechanisms during cell cycle progression, including kinases and phosphatases, with the primary kinases and phosphatase being CDKs (cyclin-dependent kinases) and PP1 (protein phosphatase 1), respectively [71,72]. PPP1R9B plays an important role in directing PP1-mediated dephosphorylation of pRb, as evidenced by its downregulation reducing PP1 activity towards pRb [73]. CYTH4’s potentially promoting ccRCC cell proliferation, likely via interaction with PPP1R9B, is intriguing, given the significant elevation of hyperphosphorylated pRb in 786-0 CYTH4 cells compared to 786-0 EV cells (Figure 3k). However, the concept of interaction with PPP1R9B contributing to CYTH4’s action in promoting 786-0 ccRCC cell proliferation remains preliminary, given that the current study did not experimentally examine this concept; for instance, the impacts of this interaction on PPP1R9B’s regulation of PP1 activities in dephosphorylating pRb, the contributions of PPP1R9B to CYTH4-promoted cell proliferation, and the structural features involved in the interaction between CYTH4 and PPP1R9B were not investigated. These aspects should be thoroughly studied in the future.

Additionally, the potential impact of CYTH4 on ccRCC immune evasion and proliferation might occur indirectly through its affecting gene expression, a concept that is supported by the observed CYTH4 correlated genes in primary ccRCC and DEGs resulting in 786-0 CYTH4 tumors. Given that CYTH4 is not a transcriptional factor, its effects on gene expression are likely attributable to changes in cell biology, for instance, cell proliferation.

Based on functional and clinical analyses, CYTH4’s ccRCC oncogenic actions might occur in part via enhancing tumor immune escape, yet the utilization of NOD/SCID mice is a limitation. To fully investigate this potential, intact mice and immunodeficient mice with humanized immune systems should be used in future studies. Given the study’s translational focus, this research lacks in-depth mechanistic details. Nonetheless, we provide the first study of CYTH4 as a novel ccRCC promoter.

## 5. Conclusions

We demonstrated for the first time that CYTH4 is a novel oncogenic factor of ccRCC. As a member of the cytohesin family, CYTH4 at the cell membrane mediates ARF6 activation [12,19,21]. Notably, we showed (1) a significant upregulation of CYTH4 in ccRCC, (2) CYTH4 expression in ccRCC tumor cells, and (3) the clear presence of CYTH4 on the plasma membrane, implying that CYTH4 is functionally upregulated in ccRCC. We confirmed this functionality; overexpression of CYTH4 promoted 786-0 ccRCC cells to form colonies in vitro and tumors in NOD/SCID mice. CYTH4 enhances 786-0 ccRCC cell proliferation likely via binding to PPP1R9B, a regulatory subunit of protein phosphatase 1 (PP1) that dephosphorylates pRb (retinoblastoma protein) and thus maintains pRb in the functional or hypophosphorylated state. The interaction between CYTH4 and PPP1R9B was associated with pRb hyperphosphorylation and the enhancement of 786-0 ccRCC cell proliferation in vitro and in vivo, suggesting a potential mechanism: CYTH4 might inhibit PPP1R9B function via physical association, leading to inactivation of pRb and subsequent cell proliferation. From the CYTH4 network, a multigene panel, SigCYTH4NW, was constructed. In retrospective studies, SigCYTH4NW correlates with cancer’s immune-suppressive features at high levels and robustly stratifies poor prognosis of ccRCC and across a panel of human cancers, suggesting that SigCYTH4NW may be used to assess the aggressiveness of ccRCC and multiple cancer types, possibly via reflecting cancer’s immune-suppressive features.

## Data Availability

All materials used in this study are either included in the Supplementary Materials or available upon request.

## Supplementary Materials

The following supporting information can be downloaded at https://www.mdpi.com/article/10.3390/biom16060923/s1: Figure S1: CYTH4 DEGs derived from 786-0 xenografts; Figure S2: CYTH4 interacting proteins; Figure S3: Analysis of CYTH4 DEGs; Figure S4: Analysis of the overlap DEGs; Figure S5: Single cell expression of Sig27 genes in human kidney; Figure S6: Analysis of the overlapped DEGs; Figure S7: Analysis of SigCYTH4NW- and CYTH4-derived stratification of poor OS; Figure S8: Single cell expression of Sig27 genes in human kidney. Table S1: RT-PCR primer; Table S2: Differential expression gene from 786-0 CYTH4 and 786-0 EV tumors; Table S3: SigCYTH4NW component genes and their involvement in ccRCC; Table S4: Genes with a correlation with CYTH4 at R ≥ 0.71; Table S5: The 10 overlapped genes and their functions; Table S6: the leading-edge gene from Hallmark MYC Target (V1/V2) and E2F Target gene sets used to create proliferation signatures. File S1: WB original images.

## References

[1] Escudier B., Porta C., Schmidinger M., Rioux-Leclercq N., Bex A., Khoo V., Grunwald V., Gillessen S., Horwich A., ESMO Guidelines Committee (2019). Renal cell carcinoma: Esmo clinical practice guidelines for diagnosis, treatment and follow-updagger. Ann. Oncol..

[2] Kim S.H., Park B., Hwang E.C., Hong S.H., Jeong C.W., Kwak C., Byun S.S., Chung J. (2019). Retrospective multicenter long-term follow-up analysis of prognostic risk factors for recurrence-free, metastasis-free, cancer-specific, and overall survival after curative nephrectomy in non-metastatic renal cell carcinoma. Front Oncol..

[3] Zbar B., Brauch H., Talmadge C., Linehan M. (1987). Loss of alleles of loci on the short arm of chromosome 3 in renal cell carcinoma. Nature.

[4] Kaelin W.G. (2002). Molecular basis of the vhl hereditary cancer syndrome. Nat. Rev. Cancer.

[5] Shirole N.H., Kaelin W.G. (2023). Von-hippel lindau and hypoxia-inducible factor at the center of renal cell carcinoma biology. Hematol. Oncol. Clin. N. Am..

[6] Liao C., Hu L., Zhang Q. (2024). Von hippel-lindau protein signalling in clear cell renal cell carcinoma. Nat. Rev. Urol..

[7] Mazumder S., Higgins P.J., Samarakoon R. (2023). Downstream targets of vhl/hif-alpha signaling in renal clear cell carcinoma progression: Mechanisms and therapeutic relevance. Cancers.

[8] Burgers F.H., van der Mijn J.C.K., Seijkens T.T.P., Jedema I., Bex A., Haanen J. (2025). Immunological features of clear-cell renal-cell carcinoma and resistance to immune checkpoint inhibitors. Nat. Rev. Nephrol..

[9] Chowdhury N., Drake C.G. (2020). Kidney cancer: An overview of current therapeutic approaches. Urol. Clin. N. Am..

[10] Meacci E., Tsai S.C., Adamik R., Moss J., Vaughan M. (1997). Cytohesin-1, a cytosolic guanine nucleotide-exchange protein for adp-ribosylation factor. Proc. Natl. Acad. Sci. USA.

[11] Cherfils J., Menetrey J., Mathieu M., Le Bras G., Robineau S., Beraud-Dufour S., Antonny B., Chardin P. (1998). Structure of the sec7 domain of the arf exchange factor arno. Nature.

[12] Das S., Malaby A.W., Nawrotek A., Zhang W., Zeghouf M., Maslen S., Skehel M., Chakravarthy S., Irving T.C., Bilsel O. (2019). Structural organization and dynamics of homodimeric cytohesin family arf gtpase exchange factors in solution and on membranes. Structure.

[13] Mansour M., Lee S.Y., Pohajdak B. (2002). The n-terminal coiled coil domain of the cytohesin/arno family of guanine nucleotide exchange factors interacts with the scaffolding protein casp. J. Biol. Chem..

[14] Lee S.Y., Pohajdak B. (2000). N-terminal targeting of guanine nucleotide exchange factors (gef) for adp ribosylation factors (arf) to the golgi. J. Cell Sci..

[15] Nagel W., Schilcher P., Zeitlmann L., Kolanus W. (1998). The ph domain and the polybasic c domain of cytohesin-1 cooperate specifically in plasma membrane association and cellular function. Mol. Biol. Cell.

[16] Ito A., Fukaya M., Okamoto H., Sakagami H. (2022). Physiological and pathological roles of the cytohesin family in neurons. Int. J. Mol. Sci..

[17] Zhang Q., Wang Q., Wu S., Zhang J. (2020). Clinical implication and immunological characterisation of the arf-gef family member cyth4 in ovarian cancer. Autoimmunity.

[18] Kennedy B.M., Harris R.E. (2018). Cyclooxygenase and lipoxygenase gene expression in the inflammogenesis of breast cancer. Inflammopharmacology.

[19] Qiu X.F., He C.M., Zeng Y.M., Deng X.L., Liang G.L., Zhong M.X., Zou M., Xiong X.J., Zhang J.D., Ye Y. (2025). Cytohesin-4/arf6 facilitates the progression of acute myeloid leukemia through activating pik3r5/pi3k/akt pathway. iScience.

[20] Gu Y., Lin X., Dong Y., Wood G., Seidah N.G., Werstuck G., Major P., Bonert M., Kapoor A., Tang D. (2023). Pcsk9 facilitates melanoma pathogenesis via a network regulating tumor immunity. J. Exp. Clin. Cancer Res. CR.

[21] Hofmann I., Thompson A., Sanderson C.M., Munro S. (2007). The arl4 family of small g proteins can recruit the cytohesin arf6 exchange factors to the plasma membrane. Curr. Biol. CB.

[22] Neira S.V., Dong Y., Zhang T., Tang D. (2025). Sig27 stratifies prostate cancer recurrence by assessing the immunosuppressive properties of tumors. Endocr. Relat. Cancer.

[23] Gao J., Aksoy B.A., Dogrusoz U., Dresdner G., Gross B., Sumer S.O., Sun Y., Jacobsen A., Sinha R., Larsson E. (2013). Integrative analysis of complex cancer genomics and clinical profiles using the cbioportal. Sci. Signal..

[24] Chandrashekar D.S., Bashel B., Balasubramanya S.A.H., Creighton C.J., Ponce-Rodriguez I., Chakravarthi B., Varambally S. (2017). Ualcan: A portal for facilitating tumor subgroup gene expression and survival analyses. Neoplasia.

[25] Zhou Y., Zhou B., Pache L., Chang M., Khodabakhshi A.H., Tanaseichuk O., Benner C., Chanda S.K. (2019). Metascape provides a biologist-oriented resource for the analysis of systems-level datasets. Nat. Commun..

[26] Li T., Fan J., Wang B., Traugh N., Chen Q., Liu J.S., Li B., Liu X.S. (2017). Timer: A web server for comprehensive analysis of tumor-infiltrating immune cells. Cancer Res..

[27] Ru B., Wong C.N., Tong Y., Zhong J.Y., Zhong S.S.W., Wu W.C., Chu K.C., Wong C.Y., Lau C.Y., Chen I. (2019). Tisidb: An integrated repository portal for tumor-immune system interactions. Bioinformatics.

[28] Ogasawara M., Kim S.C., Adamik R., Togawa A., Ferrans V.J., Takeda K., Kirby M., Moss J., Vaughan M. (2000). Similarities in function and gene structure of cytohesin-4 and cytohesin-1, guanine nucleotide-exchange proteins for adp-ribosylation factors. J. Biol. Chem..

[29] Dong Y., Shayegan B., Su Y., Neira S.V., Tang D. (2025). A novel multigene panel (sig27) robustly predicts poor prognosis of renal cell carcinoma via high-level associations with immunosuppressive features. BJC Rep..

[30] Brooks S.A., Brannon A.R., Parker J.S., Fisher J.C., Sen O., Kattan M.W., Hakimi A.A., Hsieh J.J., Choueiri T.K., Tamboli P. (2014). Clearcode34: A prognostic risk predictor for localized clear cell renal cell carcinoma. Eur. Urol..

[31] Wykoff C.C., Sotiriou C., Cockman M.E., Ratcliffe P.J., Maxwell P., Liu E., Harris A.L. (2004). Gene array of vhl mutation and hypoxia shows novel hypoxia-induced genes and that cyclin d1 is a vhl target gene. Br. J. Cancer.

[32] Kim H., Shim B.Y., Lee S.J., Lee J.Y., Lee H.J., Kim I.H. (2021). Loss of von hippel-lindau (vhl) tumor suppressor gene function: Vhl-hif pathway and advances in treatments for metastatic renal cell carcinoma (rcc). Int. J. Mol. Sci..

[33] Hu J., Tan P., Ishihara M., Bayley N.A., Schokrpur S., Reynoso J.G., Zhang Y., Lim R.J., Dumitras C., Yang L. (2023). Tumor heterogeneity in vhl drives metastasis in clear cell renal cell carcinoma. Signal Transduct. Target. Ther..

[34] Wang H., Xiao Y., Zhou W., Li Y. (2024). Integrated analysis and validation reveal cyth4 as a potential prognostic biomarker in acute myeloid leukemia. Oncol. Lett..

[35] Peng K., Zhao X., Fu Y.X., Liang Y. (2025). Eliciting antitumor immunity via therapeutic cancer vaccines. Cell. Mol. Immunol..

[36] Chen R., Zou J., Liu J., Kang R., Tang D. (2025). Damps in the immunogenicity of cell death. Mol. Cell.

[37] Perl M., Fante M.A., Herfeld K., Scherer J.N., Poeck H., Thiele Orberg E. (2025). Microbiota-derived metabolites: Key modulators of cancer immunotherapies. Med.

[38] Shultz L.D., Schweitzer P.A., Christianson S.W., Gott B., Schweitzer I.B., Tennent B., McKenna S., Mobraaten L., Rajan T.V., Greiner D.L. (1995). Multiple defects in innate and adaptive immunologic function in nod/ltsz-scid mice. J. Immunol..

[39] Leng S., Ren Y., Tian Y., Zhao W., Mou Y., Chen X., Zhou H., Wang W. (2025). Innate immunity in tumors: Roles and therapeutic targets. Front. Immunol..

[40] Zhang Y., Xue W., Xu C., Nan Y., Mei S., Ju D., Wang S., Zhang X. (2023). Innate immunity in cancer biology and therapy. Int. J. Mol. Sci..

[41] Mantovani A., Ponzetta A., Inforzato A., Jaillon S. (2019). Innate immunity, inflammation and tumour progression: Double-edged swords. J. Intern. Med..

[42] Cairns P. (2010). Renal cell carcinoma. Cancer Biomark..

[43] Zhang Y., Zhang S., Sun H., Xu L. (2025). The pathogenesis and therapeutic implications of metabolic reprogramming in renal cell carcinoma. Cell Death Discov..

[44] Jurikova M., Danihel L., Polak S., Varga I. (2016). Ki67, pcna, and mcm proteins: Markers of proliferation in the diagnosis of breast cancer. Acta Histochem..

[45] Milde-Langosch K., Karn T., Muller V., Witzel I., Rody A., Schmidt M., Wirtz R.M. (2013). Validity of the proliferation markers ki67, top2a, and racgap1 in molecular subgroups of breast cancer. Breast Cancer Res. Treat..

[46] Huttlin E.L., Bruckner R.J., Paulo J.A., Cannon J.R., Ting L., Baltier K., Colby G., Gebreab F., Gygi M.P., Parzen H. (2017). Architecture of the human interactome defines protein communities and disease networks. Nature.

[47] Schweppe D.K., Huttlin E.L., Harper J.W., Gygi S.P. (2018). Bioplex display: An interactive suite for large-scale ap-ms protein-protein interaction data. J. Proteome Res..

[48] Beenstock J., Sicheri F. (2021). The structural and functional workings of keops. Nucleic Acids Res..

[49] Barnhart-Dailey M.C., Foltz D.R. (2014). Centromere licensing: Mis18 is required to polo-ver. Curr. Biol. CB.

[50] Huang N., Xia Y., Zhang D., Wang S., Bao Y., He R., Teng J., Chen J. (2017). Hierarchical assembly of centriole subdistal appendages via centrosome binding proteins ccdc120 and ccdc68. Nat. Commun..

[51] Verdugo-Sivianes E.M., Carnero A. (2023). Spinophilin: A multiplayer tumor suppressor. Genes Dis..

[52] Ghatalia P., Rathmell W.K. (2018). Systematic review: Clearcode 34—A validated prognostic signature in clear cell renal cell carcinoma (ccrcc). Kidney Cancer.

[53] Cancer Genome Atlas Research (2013). Comprehensive molecular characterization of clear cell renal cell carcinoma. Nature.

[54] Kim B.H., Chee J.D., Bradfield C.J., Park E.S., Kumar P., MacMicking J.D. (2016). Interferon-induced guanylate-binding proteins in inflammasome activation and host defense. Nat. Immunol..

[55] Sun X., Jin G., Zhou H., Wang Y., Dai F., Zhou G. (2025). Role of guanylate-binding protein 5 in inflammatory diseases, immune diseases, cancers, and its potential therapeutic implications. Inflammopharmacology.

[56] Morad G., Helmink B.A., Sharma P., Wargo J.A. (2021). Hallmarks of response, resistance, and toxicity to immune checkpoint blockade. Cell.

[57] Kats-Ugurlu G., Oosterwijk E., Muselaers S., Oosterwijk-Wakka J., Hulsbergen-van de Kaa C., de Weijert M., van Krieken H., Desar I., van Herpen C., Maass C. (2014). Neoadjuvant sorafenib treatment of clear cell renal cell carcinoma and release of circulating tumor fragments. Neoplasia.

[58] Gu L., Li H., Gao Y., Ma X., Chen L., Li X., Zhang Y., Fan Y., Zhang X. (2015). The association of platelet count with clinicopathological significance and prognosis in renal cell carcinoma: A systematic review and meta-analysis. PLoS ONE.

[59] Liao K., Zhang X., Liu J., Teng F., He Y., Cheng J., Yang Q., Zhang W., Xie Y., Guo D. (2023). The role of platelets in the regulation of tumor growth and metastasis: The mechanisms and targeted therapy. MedComm (2020).

[60] Galluzzi L. (2025). T cell exhaustion: Early or late in tumour progression?. Nat. Rev. Immunol..

[61] Saeed A.F. (2025). Tumor-associated macrophages: Polarization, immunoregulation, and immunotherapy. Cells.

[62] Basurto-Olvera P., Serrano H., Maldonado-Bernal C. (2025). Regulatory t cells in cancer: From immunosuppression to therapeutic targeting. Front. Immunol..

[63] Chen J., Duan Y., Che J., Zhu J. (2024). Dysfunction of dendritic cells in tumor microenvironment and immunotherapy. Cancer Commun..

[64] Han Y., Wang Y., Dong X., Sun D., Liu Z., Yue J., Wang H., Li T., Wang C. (2023). Tisch2: Expanded datasets and new tools for single-cell transcriptome analyses of the tumor microenvironment. Nucleic Acids Res..

[65] Braun D.A., Hou Y., Bakouny Z., Ficial M., Sant’ Angelo M., Forman J., Ross-Macdonald P., Berger A.C., Jegede O.A., Elagina L. (2020). Interplay of somatic alterations and immune infiltration modulates response to pd-1 blockade in advanced clear cell renal cell carcinoma. Nat. Med..

[66] Tannir N.M., Albiges L., McDermott D.F., Burotto M., Choueiri T.K., Hammers H.J., Barthelemy P., Plimack E.R., Porta C., George S. (2024). Nivolumab plus ipilimumab versus sunitinib for first-line treatment of advanced renal cell carcinoma: Extended 8-year follow-up results of efficacy and safety from the phase iii checkmate 214 trial. Ann. Oncol..

[67] Yang R., Sun L., Li C.F., Wang Y.H., Yao J., Li H., Yan M., Chang W.C., Hsu J.M., Cha J.H. (2021). Galectin-9 interacts with pd-1 and tim-3 to regulate t cell death and is a target for cancer immunotherapy. Nat. Commun..

[68] Fu H., Liu Y., Xu L., Liu W., Fu Q., Liu H., Zhang W., Xu J. (2015). Galectin-9 predicts postoperative recurrence and survival of patients with clear-cell renal cell carcinoma. Tumour Biol..

[69] Jikuya R., Kishida T., Sakaguchi M., Yokose T., Yasui M., Hashizume A., Tatenuma T., Mizuno N., Muraoka K., Umemoto S. (2020). Galectin-9 expression as a poor prognostic factor in patients with renal cell carcinoma. Cancer Immunol. Immunother. CII.

[70] Andrzejczak A., Tupikowski K., Tomkiewicz A., Malkiewicz B., Ptaszkowski K., Domin A., Szydelko T., Karabon L. (2023). The variations’ in genes encoding tim-3 and its ligand, galectin-9, influence on ccrcc risk and prognosis. Int. J. Mol. Sci..

[71] Rubin S.M. (2013). Deciphering the retinoblastoma protein phosphorylation code. Trends Biochem Sci..

[72] Kim S., Leong A., Kim M., Yang H.W. (2022). Cdk4/6 initiates rb inactivation and cdk2 activity coordinates cell-cycle commitment and g1/s transition. Sci. Rep..

[73] Verdugo-Sivianes E.M., Navas L., Molina-Pinelo S., Ferrer I., Quintanal-Villalonga A., Peinado J., Garcia-Heredia J.M., Felipe-Abrio B., Munoz-Galvan S., Marin J.J. (2017). Coordinated downregulation of spinophilin and the catalytic subunits of pp1, ppp1ca/b/c, contributes to a worse prognosis in lung cancer. Oncotarget.

