# Supplementary material for: CYTH4 Facilitates Renal Cell Carcinoma via Enhancing Proliferation and Likely Immune Evasion
Source: Biomolecules. 2026 Jun 22;16(6):923. doi: 10.3390/biom16060923 (PMC13296548; doi:10.3390/biom16060923)
Supplement: Supplementary file 1 [file biomolecules-16-00923-s001.zip › Sup Figuress S1-S8.pdf]

Supplementary Figure S1

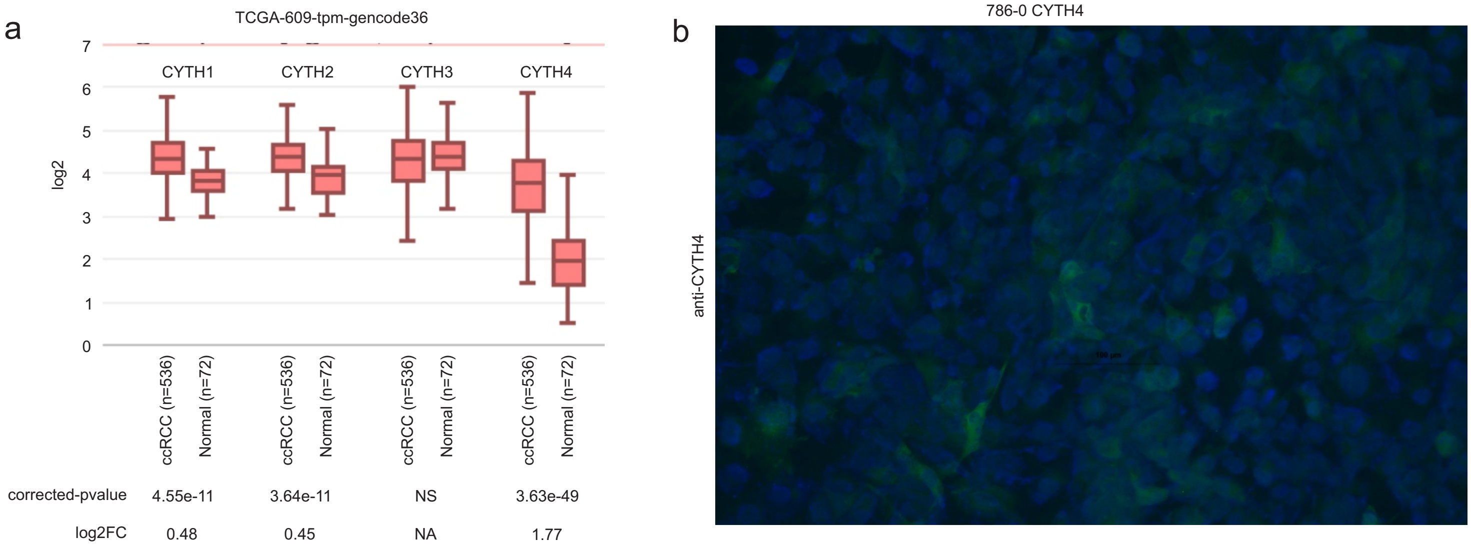

Supplementary Fig. 1 CYTH4 expression in ccRCC. (**a**) CYTH1, CYTH2, CYTH3, and CYTH4 mRNA expression was analyzed using the TCGA dataset. Analysis was performed using the R2: Genomics Analysis and Visualization Platform (http://r2.amc.nl http://r2platform.com). Statistical analysis was provided by the R2 platform. NS: not significant; NA: not available. (**b**) Immunofluorescence staining of CYTH4 in 786-0 CYTH4 cells. Scale bar: 100µm.

Supplementary Figure S2

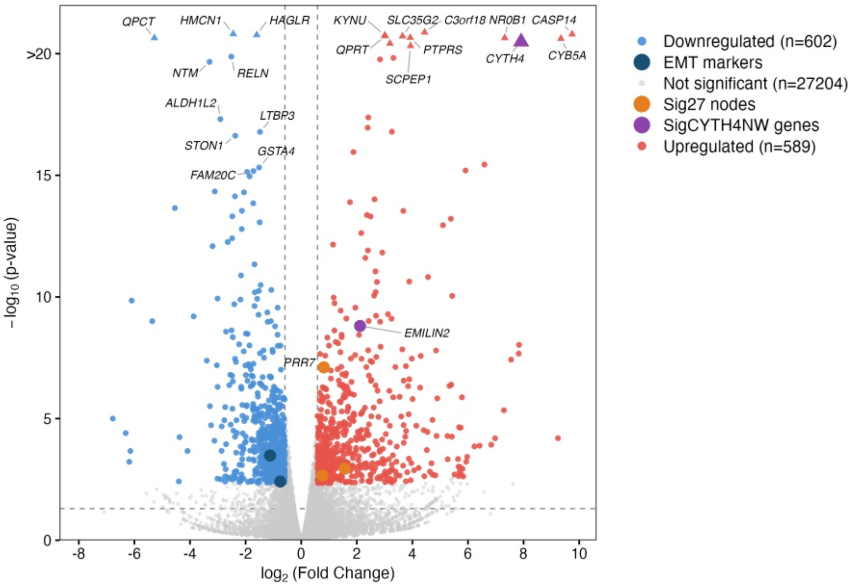

Supplementary Fig. 2 CYTH4 DEGs derived from 786-0 xenografts. Volcano plot of differentially expressed genes (DEGs) in CYTH4-overexpressing versus EV xenograft tumors derived from RNA-seq reads. Genes were defined as differentially expressed at $|\log_2 FC| > 0.585$ (1.5-fold change) and adjusted p-value < 0.05. Upregulated (n=589, red) and downregulated (n=602, blue) genes are shown. Key genes are highlighted: CYTH4 and EMILIN2 (SigCYTH4NW components, purple), Sig27 network nodes (NOD2, HDAC9, PRR7, orange). Genes exceeding the y-axis cap ($-\log_{10}$ p-value > 20) are shown as triangles.

Supplementary Figure S3

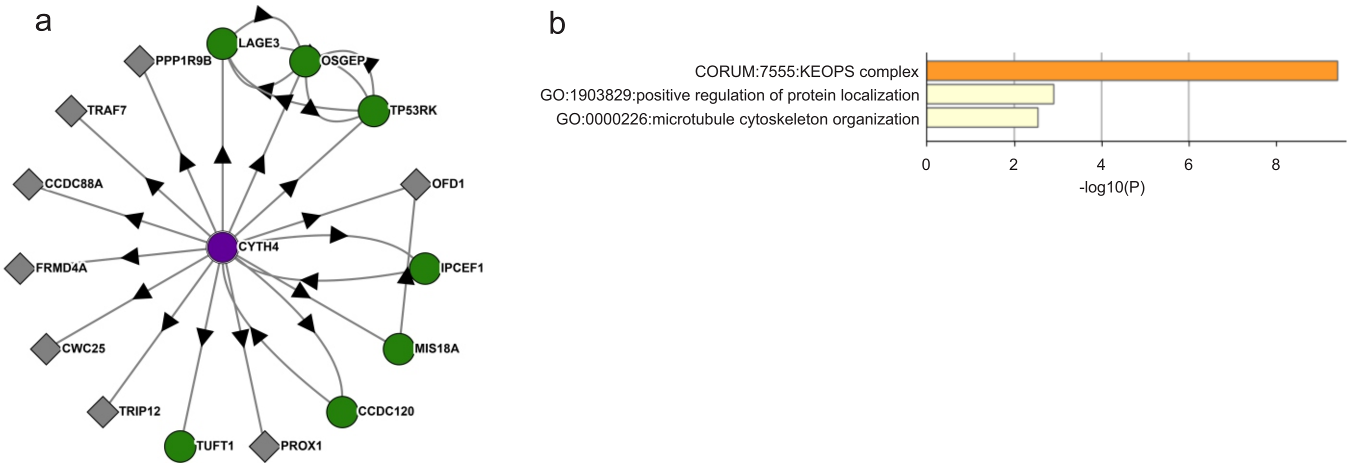

Supplementary Fig. 3 CYTH4 interacting proteins. (**a**) The BioPlex network (https://bioplex.hms.harvard.edu/explorer/network.php#) was to obtain these interactions. In brief, the relevant stable HEK293T and HCT116 cells lines were used to produce these interactions using affinity purification mass spectrometry (Schweppe et al., Journal of Proteome Research 17, 722-726, 2018). Purple circle: quered protein; Green circle: bait protein; Square: prey protein. (**b**) Enrichment of these proteins was conducted using the Metascape platform.

Supplementary Figure S4

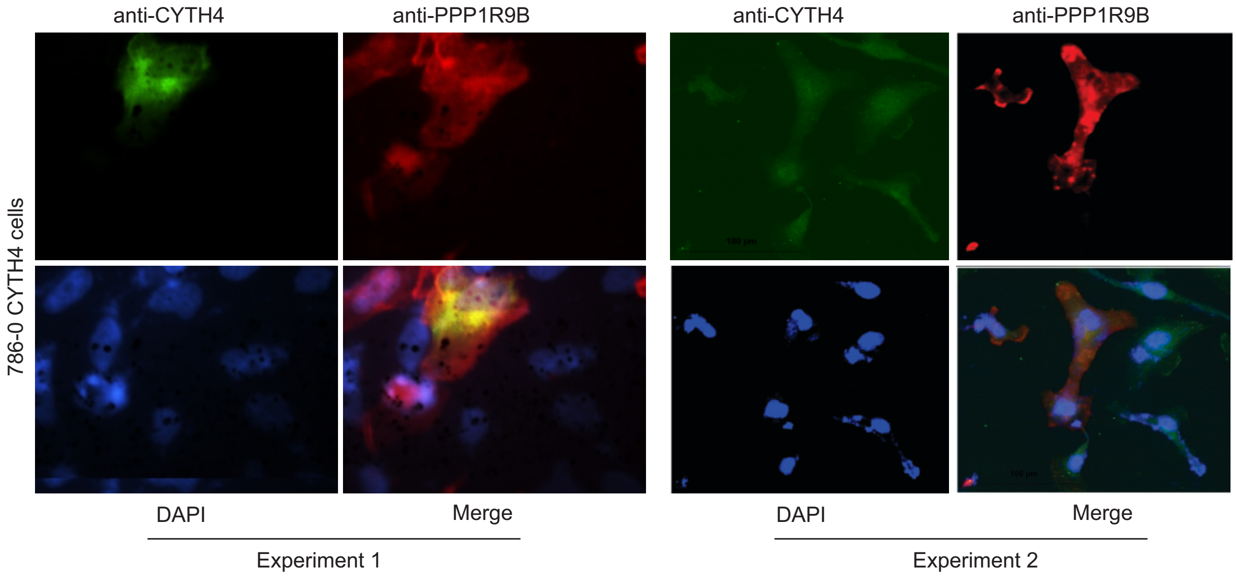

Supplementary Fig. 4 Co-localization of CYTH4 and PPP1R9B in 786-0 CYTH4 cells. IF staining for CYTH4 and PPP1R9B in the indicated cells. Images for two separate experiments are shown

Supplementary Figure S5

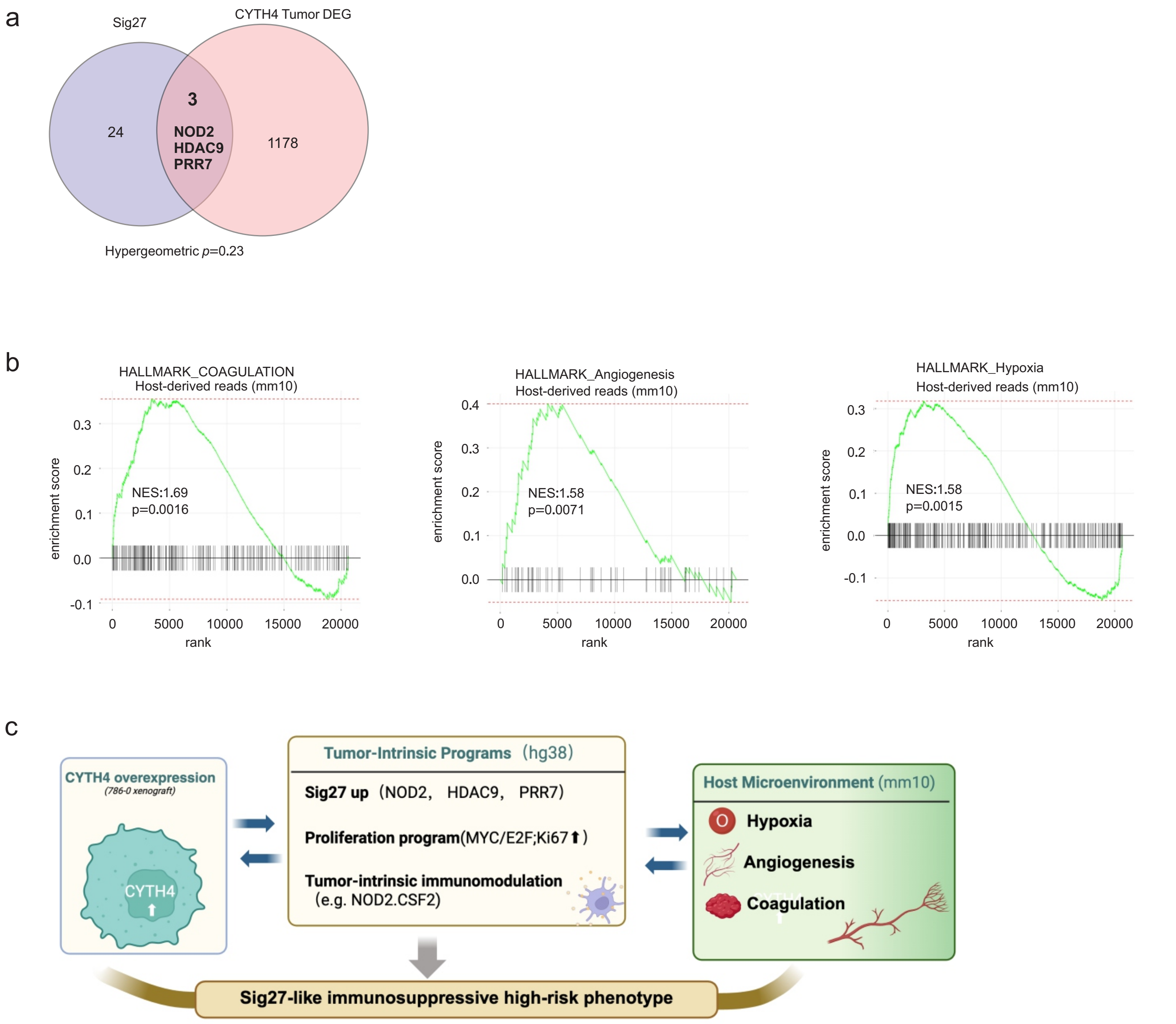

Supplementary Fig. 5 Analysis of CYTH4 DEGs. (**a**) Overlap analysis between CYTH4 DEGs (hg38 reads) and the 27-gene Sig27 panel. Three Sig27 genes (NOD2, HDAC9, PRR7) were shared between the two sets (hypergeometric p = 0.23). These overlapping genes represent key functional nodes within the Sig27 network. (**b**) GSEA of host-derived (mm10) reads reveals significant enrichment of Coagulation (NES = 1.69, p = 0.0016), Angiogenesis (NES = 1.58, p = 0.0071), and Hypoxia (NES = 1.58, p = 0.0015) Hallmark gene sets in CYTH4-overexpressing xenografts. (**c**) Schematic summary of transcriptional programs activated by CYTH4 overexpression in 786-0 xenografts. Tumor-intrinsic programs (hg38-derived reads) include upregulation of Sig27 component genes (NOD2, HDAC9, PRR7), activation of a MYC/E2F-driven proliferation program (evidenced by elevated Ki67), and induction of tumor-intrinsic immunomodulatory signaling (e.g., NOD2, CSF2). Host microenvironmental responses (mm10-derived reads) are characterized by enrichment of Hypoxia, Angiogenesis, and Coagulation hallmark pathways.

Supplementary Figure 6

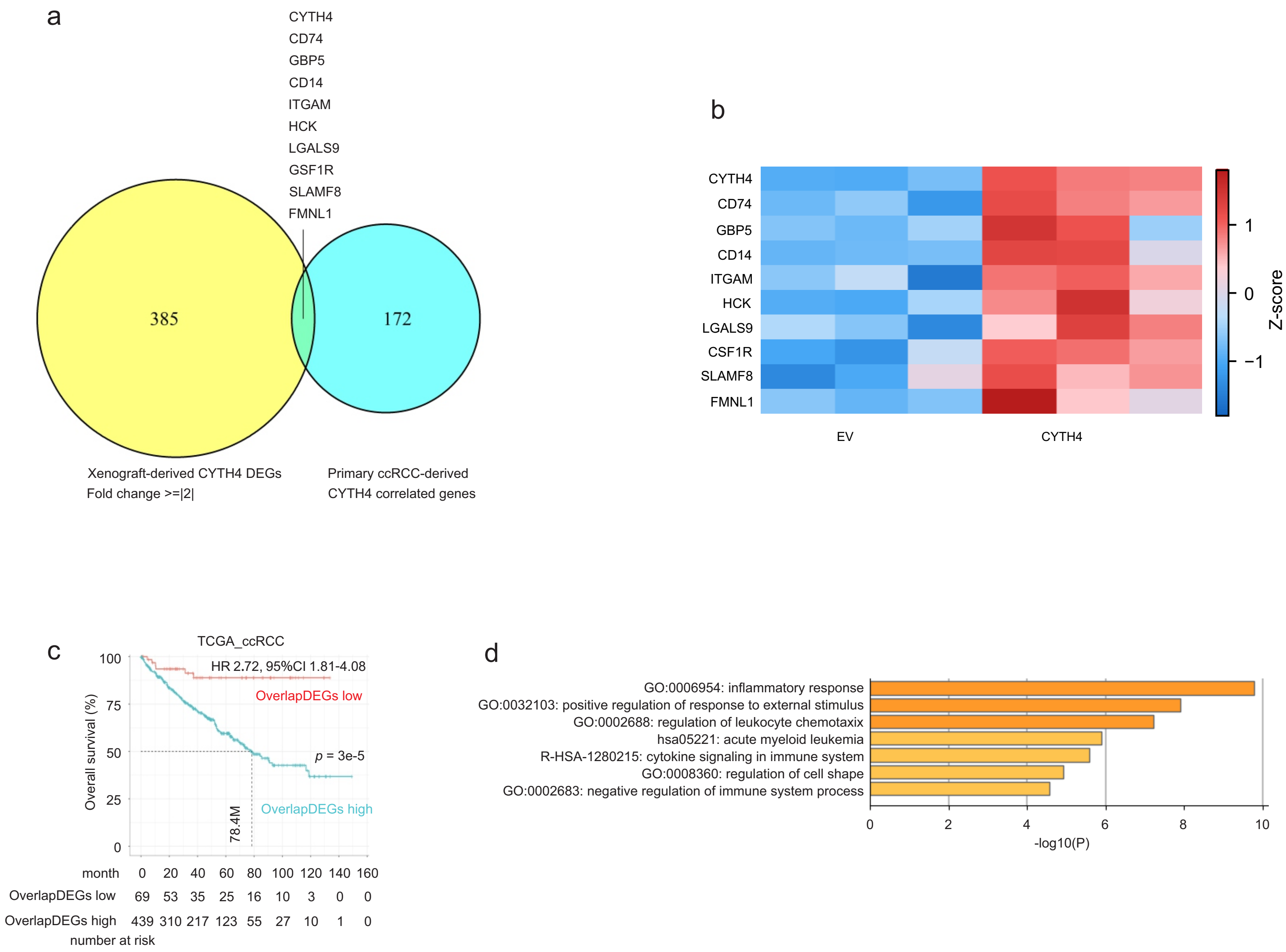

Supplementary Fig. 6 Analysis of the overlapped DEGs. (**a**) Venn Diagram for the CYTH4 DEGs (fold change ≥ 2) produced in xenografts and CYTH4 correlated genes obtained from ccRCC within the TCGA dataset. (**b**) Expression of the 10 overlapped genes in 786-0 EV and 786-0 CYTH4 tumors. Heatmap was produced using our RNA seq data. (**c**) OverlapDEGs risk scores were computed. Cutpoints were estimated using Maxstat. Kaplan-Meier survival curves and log-rank test were performed using the R survival package. (**d**) Enrichment of the 10 overlap genes was obtained using the Metascape platform.

Supplementary Figure S7

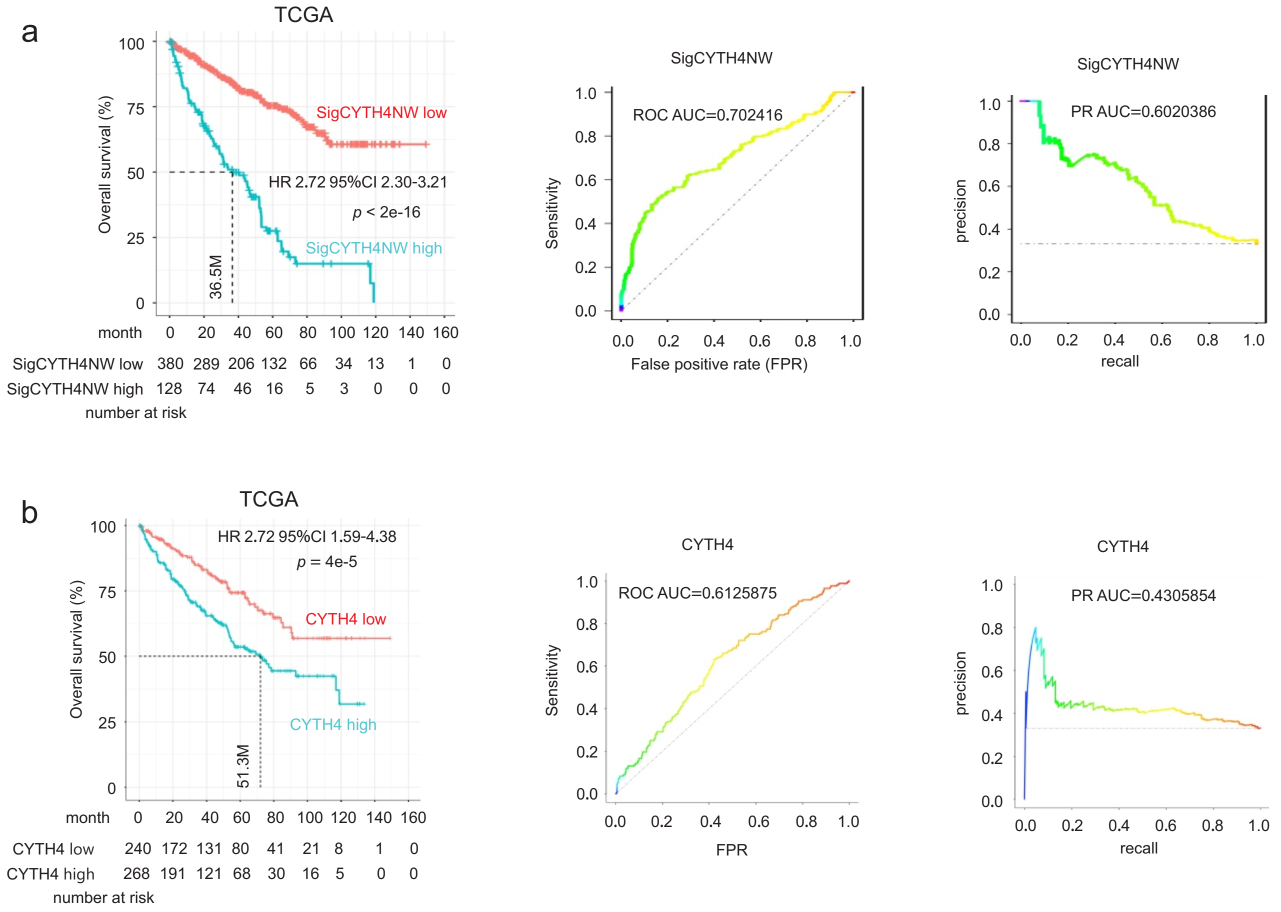

Supplementary Fig. 7 Analysis of SigCYTH4NW- and CYTH4-derived stratification of poor OS. Stratification of the OS probability in the TCGA ccRCC dataset using SigCYTH4NW (**a**) and CYTH4 (**b**). Kaplan-Meier survival, ROC AUC (Area Under the Receiver Operating Characteristic Curve), and PR AUC (Area Under the Precision-Recall Curve) curves are shown.

Supplementary Figure 8

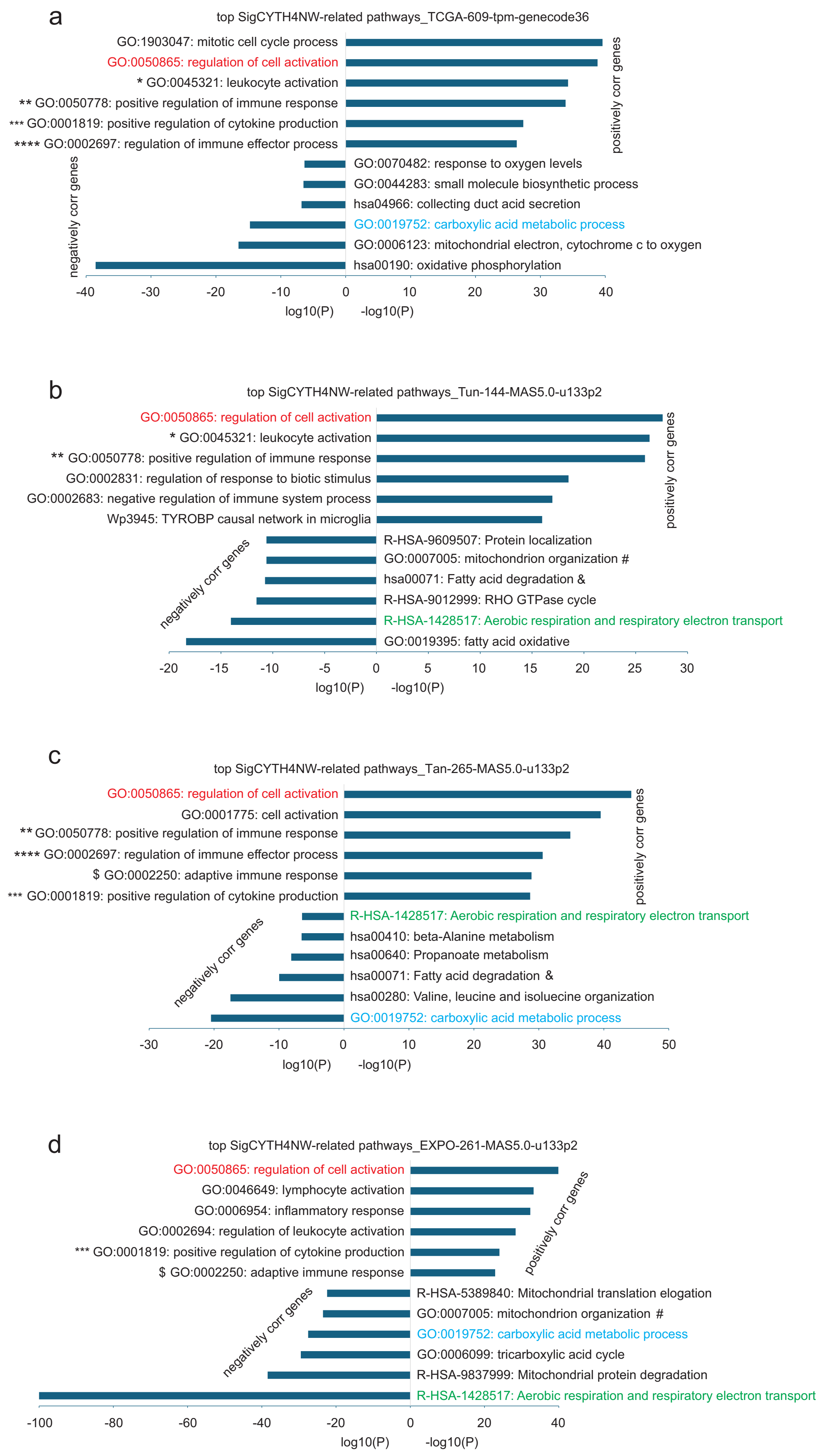

Supplementary Fig. 8 Single cell expression of Sig27 genes in human kidney. (a-d) Genes correlated with SigCYTH4NW metagene were obtained from the indicated datasets with R2. Top positively and negative correlated genes were analyzed for pathway enrichment using Metascape. The top 6 enrichments are graphed. Pathways marked with color, *, **, ***, ****, #, and & are present in more than one dataset.
