# Supplementary figures and images for: CYTH4 Facilitates Renal Cell Carcinoma via Enhancing Proliferation and Likely Immune Evasion
Source: Biomolecules. 2026 Jun 22;16(6):923. doi: 10.3390/biom16060923 (PMC13296548; doi:10.3390/biom16060923)

Uncropped Western blot images for Figs 1j, 3i, and 3k

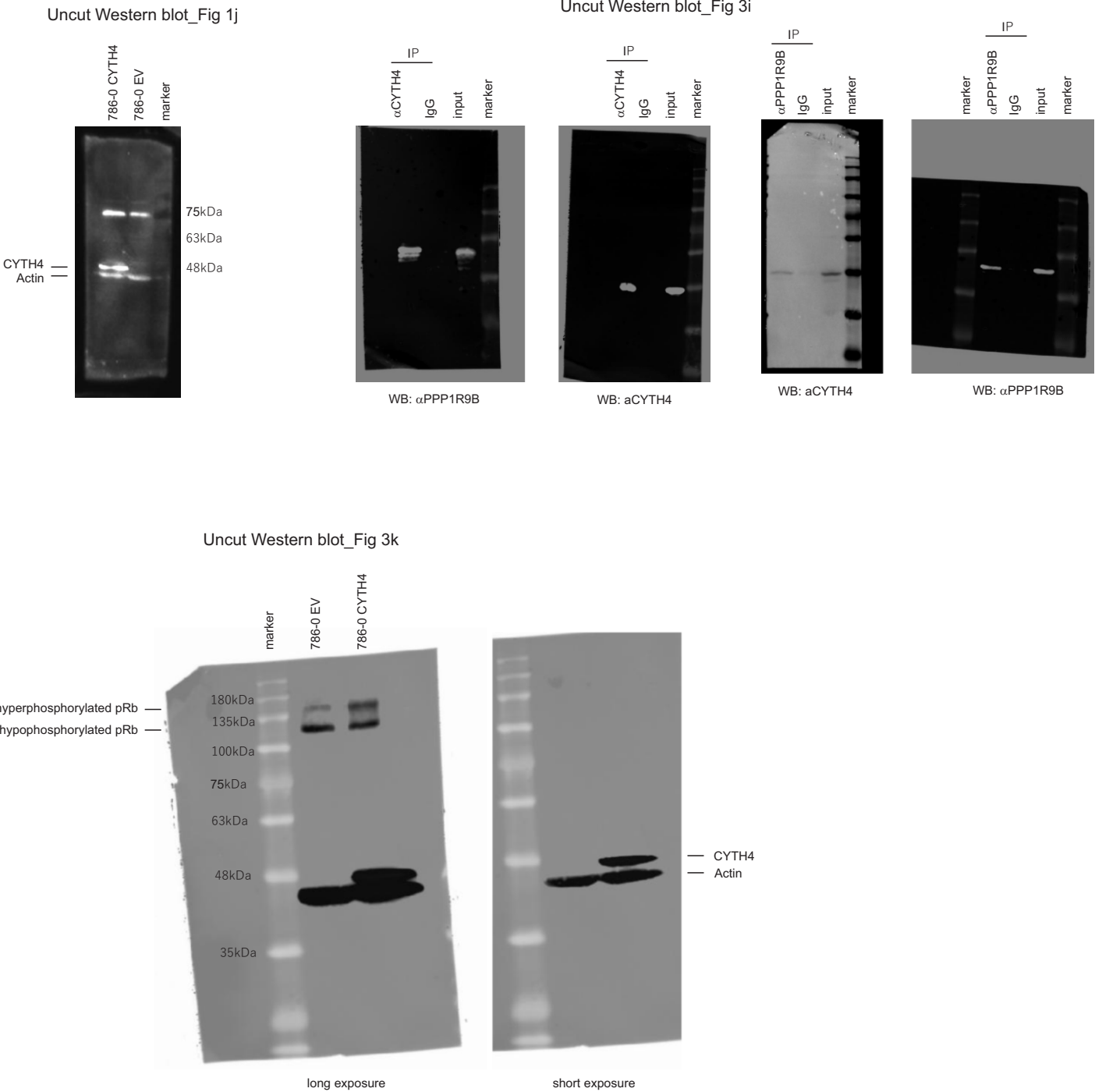

Supplement: Supplementary file 1 [file biomolecules-16-00923-s001.zip › File S1 WB original images.pdf]
